# Supplementary material for: Females exhibit smaller volumes of brain activation and lower inter-subject variability during motor tasks
Source: Sci Rep. 2023 Oct 17;13:17698. doi: 10.1038/s41598-023-44871-4 (PMC10582116; doi:10.1038/s41598-023-44871-4)
Supplement: Supplementary file 1 — Supplementary Information. [file 41598_2023_44871_MOESM1_ESM.docx]

# Primary data tables in MNI-space

**Table S1.** Jaws, Males > Females

|  |  | Max Intensity | | | Center of Gravity | | |
| --- | --- | --- | --- | --- | --- | --- | --- |
| Voxels | **Z-Max** | **X** | **Y** | **Z** | **X** | **Y** | **Z** |
| 2070 | 4.89 | 10 | -6 | 38 | 0.361 | -34.3 | 53.7 |
| 143 | 3.93 | -12 | -22 | 78 | -14.9 | -24.4 | 66.7 |
| 80 | 5.03 | 34 | -32 | 22 | 43.3 | -34.3 | 18.2 |
| 33 | 3.98 | 30 | -20 | 62 | 29.3 | -21 | 58.8 |
| 33 | 3.97 | 30 | -10 | 42 | 33.5 | -9.3 | 46.1 |
| 28 | 4.97 | 26 | -74 | 42 | 27.6 | -73.1 | 41.1 |
| 22 | 3.91 | 44 | -26 | 14 | 46.2 | -25.2 | 14 |
| 18 | 4.58 | 20 | -80 | 46 | 19.8 | -79.1 | 49.1 |
| 17 | 3.37 | 10 | -52 | 72 | 11.8 | -52.3 | 73.4 |
| 17 | 3.83 | 16 | -70 | 30 | 14.5 | -71.1 | 32.7 |
| 15 | 3.83 | -12 | -12 | 62 | -9.7 | -10.8 | 63.7 |
| 9 | 3.96 | 36 | -76 | 44 | 35 | -77.3 | 43.8 |
| 8 | 3.59 | -8 | -18 | 62 | -7.26 | -19.2 | 61.8 |
| 6 | 3.82 | 12 | -78 | 46 | 13.3 | -77.4 | 47 |
| 6 | 4.53 | -16 | -82 | 44 | -15.4 | -80.7 | 43.4 |
| 4 | 3.71 | 46 | -16 | 46 | 46.9 | -16.5 | 45.5 |
| 4 | 3.68 | 6 | -72 | 40 | 5.48 | -72.6 | 39.5 |
| 4 | 3.46 | 46 | -64 | 48 | 46 | -65.5 | 47.5 |
| 4 | 3.15 | 38 | -18 | 58 | 38.5 | -18 | 56.5 |
| 3 | 3.78 | 10 | -64 | 32 | 10 | -63.3 | 32 |
| 3 | 3.09 | -18 | -14 | 64 | -18 | -12.7 | 63.4 |
| 2 | 4.09 | 22 | -56 | 20 | 22 | -56 | 19 |
| 2 | 4.05 | 22 | -66 | 38 | 22 | -65 | 37 |
| 2 | 3.77 | -12 | -66 | 28 | -12 | -66 | 29 |
| 1 | 3.41 | 0 | -40 | 38 | 0 | -40 | 38 |
| 1 | 4.04 | 34 | -26 | 42 | 34 | -26 | 42 |
| 1 | 3.8 | 40 | -52 | 38 | 40 | -52 | 38 |
| 1 | 3.87 | 40 | -50 | 48 | 40 | -50 | 48 |
| 1 | 3.33 | 8 | -78 | 50 | 8 | -78 | 50 |
| 1 | 4.37 | 40 | -56 | 18 | 40 | -56 | 18 |
| 1 | 3.01 | 30 | -18 | 50 | 30 | -18 | 50 |
| 1 | 3.72 | 50 | 2 | 52 | 50 | 2 | 52 |
| 1 | 3.79 | 48 | 2 | 56 | 48 | 2 | 56 |
| 1 | 3.9 | 6 | -70 | 26 | 6 | -70 | 26 |
| 1 | 4.97 | 44 | -48 | 62 | 44 | -48 | 62 |
| 1 | 2.54 | 6 | -52 | 68 | 6 | -52 | 68 |

**Table S2.** Tongue, Males > Females

|  |  | Max Intensity | | | Center of Gravity | | |
| --- | --- | --- | --- | --- | --- | --- | --- |
| Voxels | **Z-Max** | **X** | **Y** | **Z** | **X** | **Y** | **Z** |
| 128 | 4.6 | -16 | -24 | 68 | -13.1 | -25.4 | 66.7 |
| 48 | 3.74 | 2 | -48 | 42 | 4.51 | -48.9 | 44.9 |
| 47 | 4.83 | 18 | -24 | 66 | 15.5 | -24.2 | 68.3 |
| 19 | 3.85 | 8 | -38 | 52 | 11.7 | -38.5 | 57.5 |
| 15 | 4.19 | 18 | -32 | 40 | 15.9 | -32 | 42.1 |
| 15 | 4.46 | 20 | -80 | 46 | 22.8 | -79.6 | 47.9 |
| 14 | 3.92 | 46 | -16 | 44 | 46.3 | -17.1 | 46.2 |
| 9 | 4.82 | 26 | -74 | 42 | 26.5 | -73.1 | 40.8 |
| 4 | 3.6 | -4 | -32 | 40 | -3.01 | -32.4 | 40.5 |
| 3 | 3.94 | -12 | -32 | 50 | -12 | -32.6 | 49.4 |
| 2 | 4.14 | 34 | -82 | 24 | 34 | -81 | 24 |
| 2 | 3.88 | 14 | -90 | 34 | 13 | -90 | 33 |
| 2 | 4.31 | 24 | -66 | 38 | 24 | -66 | 39 |
| 2 | 4.13 | -6 | -28 | 46 | -5.08 | -28 | 46 |
| 2 | 3.31 | -26 | -36 | 62 | -26 | -36 | 61 |
| 2 | 4.51 | 44 | -48 | 62 | 44 | -49 | 62 |
| 1 | 4.77 | 22 | -76 | 16 | 22 | -76 | 16 |
| 1 | 5.24 | 36 | -36 | 20 | 36 | -36 | 20 |
| 1 | 4.19 | 18 | -70 | 30 | 18 | -70 | 30 |
| 1 | 4.64 | 32 | -82 | 34 | 32 | -82 | 34 |
| 1 | 3.88 | 18 | -70 | 34 | 18 | -70 | 34 |
| 1 | 3.8 | 8 | -38 | 36 | 8 | -38 | 36 |
| 1 | 3.86 | -6 | -36 | 46 | -6 | -36 | 46 |
| 1 | 3.32 | 0 | -42 | 50 | 0 | -42 | 50 |
| 1 | 3.77 | -24 | -36 | 54 | -24 | -36 | 54 |
| 1 | 3.32 | 36 | -20 | 54 | 36 | -20 | 54 |
| 1 | 3.68 | 4 | -20 | 56 | 4 | -20 | 56 |
| 1 | 3.79 | 8 | -40 | 58 | 8 | -40 | 58 |

**Table S3.** Upper Arms, Males > Females

|  |  | Max Intensity | | | Center of Gravity | | |
| --- | --- | --- | --- | --- | --- | --- | --- |
| Voxels | **Z-Max** | **X** | **Y** | **Z** | **X** | **Y** | **Z** |
| 15 | 4.73 | 40 | -12 | 34 | 41.9 | -12.8 | 34 |
| 5 | 4.56 | 60 | 2 | 10 | 59.2 | 1.62 | 10.8 |
| 5 | 4.53 | 64 | -2 | 32 | 64 | -1.24 | 29.6 |
| 3 | 4.9 | -30 | 26 | 2 | -30.6 | 26.6 | 2 |

**Table S4.** Forearms, Males > Females

|  |  | Max Intensity | | | Center of Gravity | | |
| --- | --- | --- | --- | --- | --- | --- | --- |
| Voxels | **Z-Max** | **X** | **Y** | **Z** | **X** | **Y** | **Z** |
| 7 | 4.92 | 58 | 0 | 10 | 59.1 | 0.86 | 10.3 |

**Table S5.** Wrists, Males > Females

|  |  | Max Intensity | | | Center of Gravity | | |
| --- | --- | --- | --- | --- | --- | --- | --- |
| Voxels | **Z-Max** | **X** | **Y** | **Z** | **X** | **Y** | **Z** |
| 1660 | 5.01 | -24 | -84 | 44 | -12.9 | -65.3 | 37 |
| 607 | 5.32 | -10 | 34 | 18 | -4.18 | 44.7 | 13.5 |
| 352 | 4.83 | 46 | -8 | 30 | 52.6 | -1.45 | 20.8 |
| 336 | 4.8 | 4 | -52 | 42 | 4.75 | -40.8 | 38 |
| 229 | 4.75 | -30 | 28 | 56 | -25.3 | 28.4 | 47 |
| 210 | 4.99 | 54 | -60 | 30 | 49.8 | -58.4 | 30.9 |
| 177 | 5.03 | -28 | -6 | -6 | -31.7 | -19.5 | 1.92 |
| 166 | 4.15 | 18 | 46 | 36 | 21.4 | 32.4 | 40 |
| 160 | 4.9 | 8 | -30 | 60 | 14.9 | -26 | 64.2 |
| 94 | 4.19 | 42 | 16 | 32 | 40.5 | 12.5 | 33.6 |
| 93 | 3.92 | -40 | -74 | 28 | -39.6 | -80.6 | 32.1 |
| 74 | 3.85 | -42 | -18 | 32 | -51.6 | -10.8 | 25 |
| 73 | 3.6 | -8 | -24 | 68 | -14.8 | -26 | 64.6 |
| 65 | 4.35 | 28 | -12 | -2 | 29.3 | -4.78 | -6.95 |
| 63 | 3.87 | -38 | -64 | 12 | -39.6 | -68.4 | 16.4 |
| 51 | 3.59 | 46 | -34 | 22 | 46.4 | -30 | 19.7 |
| 33 | 4.15 | -64 | -22 | 34 | -62.7 | -23.5 | 36.5 |
| 27 | 3.69 | -32 | 44 | 12 | -36 | 45 | 16.5 |
| 27 | 3.62 | 38 | 26 | 40 | 37.7 | 27.2 | 40 |
| 26 | 4.81 | 36 | -24 | 4 | 35.7 | -22.6 | 4.71 |
| 24 | 4.59 | 38 | -42 | 18 | 42.1 | -41.9 | 17.6 |
| 21 | 4.78 | -44 | 38 | 32 | -44.5 | 36.1 | 33.6 |
| 19 | 3.88 | 12 | -32 | 48 | 11.9 | -34 | 47.3 |
| 18 | 4.12 | 2 | 62 | 28 | 2.11 | 61.2 | 32.5 |
| 18 | 3.19 | 32 | 14 | 8 | 35.2 | 12.4 | 8.23 |
| 17 | 4.05 | 32 | 40 | 46 | 31.3 | 38.3 | 47.1 |
| 16 | 3.26 | -16 | 36 | 52 | -12.7 | 36 | 55 |
| 12 | 3 | 28 | -30 | 60 | 26.3 | -31.3 | 59 |
| 9 | 3.07 | 24 | 16 | 46 | 23.6 | 16.8 | 47.5 |
| 8 | 3.8 | 4 | -10 | 6 | 3.5 | -11 | 7.45 |
| 7 | 4.21 | 22 | -82 | 46 | 21.9 | -81.2 | 46 |
| 6 | 3.25 | 48 | -8 | 10 | 49 | -8.35 | 10.6 |
| 6 | 3.65 | -34 | 14 | 46 | -33.7 | 13.3 | 47 |
| 6 | 3.37 | -38 | 4 | 50 | -38 | 5.29 | 50.3 |
| 5 | 3.39 | 68 | -12 | 4 | 68.8 | -13.2 | 3.23 |
| 5 | 4.89 | 44 | -50 | 62 | 41.6 | -50.8 | 63.2 |
| 5 | 3.28 | 2 | 46 | -8 | 2.76 | 47.1 | -6.83 |
| 4 | 3.71 | 34 | 24 | 28 | 33.1 | 23.5 | 28 |
| 4 | 4.08 | 26 | -74 | 42 | 26.4 | -73.5 | 40.5 |
| 4 | 3.63 | 24 | 32 | 54 | 25.4 | 30.6 | 55.5 |
| 4 | 3.22 | -20 | -18 | 54 | -20 | -18.5 | 55.5 |
| 4 | 3.08 | 0 | 68 | 2 | -0.49 | 68 | 1.93 |
| 4 | 2.99 | 2 | 46 | 0 | 1.49 | 47.5 | -0.974 |
| 4 | 3.94 | -66 | -6 | 12 | -66 | -6.49 | 12 |
| 4 | 3.91 | 2 | 20 | 30 | 2 | 20 | 29 |
| 4 | 4.47 | 16 | 24 | 10 | 15.6 | 22.6 | 10.4 |
| 4 | 3.91 | 34 | 18 | 26 | 33.1 | 18 | 25.1 |
| 3 | 3.14 | 62 | -14 | 12 | 62 | -14 | 12 |
| 3 | 3.9 | -64 | -2 | 8 | -63.4 | -0.722 | 7.36 |
| 3 | 2.82 | -8 | 28 | 22 | -8 | 27.3 | 22.7 |
| 3 | 2.49 | 42 | -66 | 20 | 42 | -66 | 20 |
| 3 | 3.93 | -46 | 28 | 42 | -47.3 | 26 | 40.7 |
| 3 | 4.01 | -26 | -10 | 2 | -25.4 | -8.73 | 2 |
| 3 | 3.33 | 12 | -58 | 26 | 11.4 | -58 | 26.6 |
| 3 | 3.67 | -8 | 44 | -10 | -7.34 | 45.3 | -10 |
| 3 | 2.97 | 36 | -52 | 38 | 37.9 | -52 | 38 |
| 3 | 3.15 | 22 | 22 | 62 | 23.3 | 21.4 | 62 |
| 2 | 2.84 | 24 | 16 | 40 | 24 | 17 | 40 |
| 2 | 3.91 | 46 | -22 | 22 | 46 | -21 | 22 |
| 2 | 3.73 | 26 | 42 | -16 | 26.9 | 42 | -16 |
| 2 | 4.36 | -38 | 20 | 54 | -38 | 20.9 | 54 |
| 2 | 3.6 | -4 | 18 | 20 | -4.96 | 18 | 20 |
| 2 | 3.29 | -2 | -74 | 56 | -2 | -74 | 55 |
| 2 | 3.37 | -16 | 44 | 50 | -15 | 45 | 50 |
| 2 | 3.08 | 18 | 16 | -10 | 19 | 17 | -10 |
| 2 | 2.98 | -20 | 24 | 48 | -20 | 24 | 49 |
| 2 | 3.19 | -4 | 54 | -10 | -4 | 54 | -9.09 |
| 2 | 3.68 | -18 | -44 | -6 | -17.1 | -44.9 | -5.08 |
| 2 | 3.14 | 56 | -16 | 12 | 56 | -16 | 11 |
| 2 | 2.94 | -54 | -32 | 38 | -54 | -33 | 39 |
| 2 | 3.67 | 56 | -16 | 6 | 56.9 | -16 | 6 |
| 2 | 3.5 | -44 | 6 | 34 | -43 | 6 | 34 |
| 2 | 3.8 | 42 | 24 | 6 | 42 | 24 | 6.97 |
| 1 | 3.62 | 30 | -12 | 8 | 30 | -12 | 8 |
| 1 | 3.38 | -2 | -46 | 36 | -2 | -46 | 36 |
| 1 | 3.35 | -62 | 4 | 8 | -62 | 4 | 8 |
| 1 | 3.24 | 30 | 18 | 8 | 30 | 18 | 8 |
| 1 | 3.25 | -30 | 8 | 38 | -30 | 8 | 38 |
| 1 | 3.14 | -46 | -64 | 40 | -46 | -64 | 40 |
| 1 | 3.34 | -34 | 8 | 40 | -34 | 8 | 40 |
| 1 | 3.43 | -44 | 10 | 40 | -44 | 10 | 40 |
| 1 | 2.97 | -40 | 14 | 40 | -40 | 14 | 40 |
| 1 | 3.54 | 38 | -40 | 24 | 38 | -40 | 24 |
| 1 | 4.37 | 16 | 28 | 4 | 16 | 28 | 4 |
| 1 | 2.87 | 32 | 40 | 42 | 32 | 40 | 42 |
| 1 | 3.15 | -44 | -64 | 44 | -44 | -64 | 44 |
| 1 | 2.64 | 34 | 2 | 4 | 34 | 2 | 4 |
| 1 | 3.62 | 22 | 20 | -6 | 22 | 20 | -6 |
| 1 | 3.87 | -20 | 12 | -6 | -20 | 12 | -6 |
| 1 | 3.42 | 2 | 50 | 44 | 2 | 50 | 44 |
| 1 | 3.97 | -58 | 2 | 10 | -58 | 2 | 10 |
| 1 | 4.18 | -20 | 8 | -8 | -20 | 8 | -8 |
| 1 | 2.84 | 0 | 64 | 10 | 0 | 64 | 10 |
| 1 | 4.07 | -18 | -50 | 28 | -18 | -50 | 28 |
| 1 | 3.49 | 6 | 44 | 14 | 6 | 44 | 14 |
| 1 | 3.64 | -48 | -4 | 16 | -48 | -4 | 16 |
| 1 | 3.9 | -6 | 48 | 50 | -6 | 48 | 50 |
| 1 | 3.93 | 24 | 18 | -12 | 24 | 18 | -12 |
| 1 | 2.8 | -40 | 10 | 52 | -40 | 10 | 52 |
| 1 | 3.97 | -14 | 0 | 20 | -14 | 0 | 20 |
| 1 | 3.26 | 26 | 38 | -14 | 26 | 38 | -14 |
| 1 | 3.9 | 24 | 10 | -14 | 24 | 10 | -14 |
| 1 | 3.15 | 2 | -32 | 26 | 2 | -32 | 26 |
| 1 | 3.04 | 30 | 30 | 54 | 30 | 30 | 54 |
| 1 | 3.76 | 42 | -44 | 26 | 42 | -44 | 26 |
| 1 | 3.35 | 14 | 14 | -20 | 14 | 14 | -20 |
| 1 | 3.38 | -24 | -34 | 60 | -24 | -34 | 60 |
| 1 | 3.74 | 42 | 24 | -22 | 42 | 24 | -22 |
| 1 | 5.27 | 32 | -72 | -38 | 32 | -72 | -38 |

**Table S6.** Fingers, Males > Females

|  |  | Max Intensity | | | Center of Gravity | | |
| --- | --- | --- | --- | --- | --- | --- | --- |
| Voxels | **Z-Max** | **X** | **Y** | **Z** | **X** | **Y** | **Z** |
| 2658 | 4.66 | 4 | -48 | 42 | 2.86 | -65.3 | 29.2 |
| 1294 | 4.7 | -14 | -24 | 52 | 2.45 | -27.2 | 61.8 |
| 390 | 4.55 | 46 | -52 | 28 | 47.9 | -60.2 | 24.7 |
| 229 | 4.88 | 60 | 2 | 10 | 53.5 | -1.78 | 20.5 |
| 111 | 4.06 | 46 | -40 | 16 | 47.3 | -31.4 | 17.9 |
| 55 | 3.96 | -46 | -54 | 30 | -46 | -56.9 | 27.1 |
| 41 | 4.11 | -38 | -74 | 20 | -40.6 | -69.4 | 18.8 |
| 37 | 4.82 | 36 | -22 | 6 | 36.2 | -21.6 | 3.93 |
| 35 | 4.52 | 12 | -12 | 38 | 11 | -14.3 | 38.3 |
| 33 | 3.7 | 4 | -18 | 40 | 1.64 | -19.2 | 36.1 |
| 29 | 3.43 | 32 | -10 | 46 | 33 | -8.2 | 45.5 |
| 19 | 4.69 | 64 | -2 | 28 | 63.2 | -2.09 | 29.5 |
| 18 | 4.06 | 58 | -32 | 0 | 60.3 | -30.7 | 0.834 |
| 15 | 3.39 | 34 | -74 | 32 | 34.4 | -75.5 | 35.5 |
| 15 | 4.46 | -34 | -22 | 16 | -36.5 | -22.4 | 14.1 |
| 13 | 3.94 | 44 | -50 | 62 | 42.3 | -51.6 | 62.9 |
| 13 | 3.63 | 32 | -2 | 14 | 33.7 | -1.09 | 11.1 |
| 11 | 2.93 | -26 | -82 | 44 | -24.1 | -81.4 | 42.9 |
| 7 | 3.02 | 2 | 6 | 40 | 1.72 | 6.84 | 37.7 |
| 7 | 4.1 | 38 | -34 | 44 | 37.4 | -35.2 | 44.6 |
| 7 | 3.38 | -8 | -36 | 50 | -8.22 | -34.9 | 49.8 |
| 6 | 3.66 | -52 | -66 | 34 | -51.7 | -65 | 32.4 |
| 6 | 3.41 | 0 | -12 | 36 | -1.01 | -8.39 | 34 |
| 5 | 3.91 | 64 | -14 | 12 | 60.1 | -14.4 | 10.4 |
| 4 | 3.73 | 30 | -12 | 8 | 31 | -11.5 | 9.48 |
| 3 | 3.43 | 42 | -2 | 16 | 40.7 | -2.61 | 16 |
| 3 | 3.3 | 52 | -38 | 14 | 52.6 | -38.6 | 13.4 |
| 2 | 3.59 | 40 | -68 | 44 | 40 | -68.9 | 44 |
| 2 | 3.4 | 22 | -56 | 62 | 23 | -56 | 62 |
| 2 | 3.65 | 54 | -28 | 54 | 53 | -28 | 54 |
| 2 | 3.46 | 40 | -2 | 60 | 40 | -2.99 | 60 |
| 2 | 3.86 | -38 | -22 | -4 | -39 | -22 | -4 |
| 2 | 3.29 | -10 | 0 | 34 | -10 | -0.962 | 34 |
| 2 | 2.6 | 6 | -58 | 36 | 6 | -57 | 36 |
| 1 | 2.85 | -2 | -10 | 30 | -2 | -10 | 30 |
| 1 | 3.71 | -40 | -56 | 22 | -40 | -56 | 22 |
| 1 | 2.43 | 38 | -16 | 38 | 38 | -16 | 38 |
| 1 | 4.4 | 36 | -8 | 20 | 36 | -8 | 20 |
| 1 | 2.45 | -18 | -56 | 18 | -18 | -56 | 18 |
| 1 | 3.32 | 18 | -12 | 40 | 18 | -12 | 40 |
| 1 | 3.25 | 44 | -6 | 14 | 44 | -6 | 14 |
| 1 | 3.32 | 68 | -16 | 14 | 68 | -16 | 14 |
| 1 | 2.83 | 58 | -40 | 14 | 58 | -40 | 14 |
| 1 | 3.74 | 52 | -18 | 12 | 52 | -18 | 12 |
| 1 | 4.36 | 52 | -26 | 50 | 52 | -26 | 50 |
| 1 | 4.08 | -32 | 2 | 6 | -32 | 2 | 6 |
| 1 | 3.64 | 50 | 4 | 54 | 50 | 4 | 54 |
| 1 | 3.83 | 70 | -20 | 4 | 70 | -20 | 4 |
| 1 | 3.75 | 58 | -34 | -4 | 58 | -34 | -4 |
| 1 | 4.2 | 40 | -16 | -8 | 40 | -16 | -8 |

**Table S7.** Ankles, Males > Females

|  |  | Max Intensity | | | Center of Gravity | | |
| --- | --- | --- | --- | --- | --- | --- | --- |
| Voxels | **Z-Max** | **X** | **Y** | **Z** | **X** | **Y** | **Z** |
| 157 | 5.27 | 58 | 2 | 10 | 60.1 | 2.83 | 17.9 |
| 137 | 4.45 | 42 | -16 | 62 | 39.5 | -17.6 | 50.6 |
| 100 | 4.17 | -16 | -80 | 44 | -13.8 | -78.9 | 35.3 |
| 80 | 4.39 | 30 | -22 | 58 | 27.3 | -24 | 55.4 |
| 17 | 3.5 | 38 | -2 | 62 | 41.2 | -0.763 | 58.7 |
| 14 | 3.81 | 16 | -22 | 68 | 17.8 | -23 | 65.8 |
| 10 | 3.86 | 16 | -80 | 36 | 16 | -79 | 36 |
| 8 | 3.86 | 30 | -10 | 42 | 30.7 | -8.79 | 43.7 |
| 8 | 4.51 | 38 | 6 | 60 | 35.9 | 5.06 | 58.5 |
| 7 | 3.61 | 18 | -70 | 32 | 18.3 | -70.9 | 31.4 |
| 6 | 3.89 | 52 | -2 | 24 | 51.3 | -2 | 24.3 |
| 6 | 3.49 | 60 | -8 | 32 | 57.4 | -7.05 | 30.7 |
| 2 | 4.36 | 48 | 16 | 22 | 48.9 | 16 | 22 |
| 2 | 3.82 | -10 | -64 | 32 | -10 | -64 | 31 |
| 2 | 3.81 | 16 | -24 | 56 | 16 | -25 | 56 |
| 1 | 3.07 | 62 | 12 | 0 | 62 | 12 | 0 |
| 1 | 3.06 | 66 | 2 | 12 | 66 | 2 | 12 |
| 1 | 3.84 | 22 | -70 | 26 | 22 | -70 | 26 |
| 1 | 3.91 | -6 | -84 | 34 | -6 | -84 | 34 |
| 1 | 3.63 | 18 | -66 | 36 | 18 | -66 | 36 |
| 1 | 3.17 | 50 | -10 | 36 | 50 | -10 | 36 |
| 1 | 3.82 | -4 | -82 | 38 | -4 | -82 | 38 |
| 1 | 4.19 | 28 | -70 | 40 | 28 | -70 | 40 |
| 1 | 4.53 | 20 | -80 | 46 | 20 | -80 | 46 |
| 1 | 2.77 | 30 | -20 | 46 | 30 | -20 | 46 |
| 1 | 4.78 | -16 | -60 | 70 | -16 | -60 | 70 |

**Table S8.** Right leg, Females > Males

|  |  | Max Intensity | | | Center of Gravity | | |
| --- | --- | --- | --- | --- | --- | --- | --- |
| Voxels | **Z-Max** | **X** | **Y** | **Z** | **X** | **Y** | **Z** |
| 494 | 4.56 | -54 | -22 | -22 | -52.1 | -19.9 | -16.2 |
| 95 | 4.19 | -40 | 0 | -34 | -37.9 | -1.44 | -30.1 |
| 21 | 3.38 | -42 | -10 | -30 | -45.4 | -10.3 | -28.6 |
| 8 | 3.78 | -60 | -6 | -4 | -59 | -8.39 | -4.25 |
| 4 | 4.55 | 44 | -22 | -14 | 42.6 | -24.9 | -12.6 |
| 2 | 3.22 | -58 | -8 | -10 | -58 | -8.99 | -10 |
| 1 | 3.03 | -46 | -6 | -36 | -46 | -6 | -36 |
| 1 | 4.09 | 40 | -20 | -14 | 40 | -20 | -14 |
| 1 | 2.9 | -52 | -18 | -10 | -52 | -18 | -10 |
| 1 | 3.34 | -62 | -8 | -10 | -62 | -8 | -10 |
| 1 | 4.7 | 46 | -48 | 4 | 46 | -48 | 4 |

**Analysis of motion parameters**

Here we show data tables from linear mixed effects models to assess between sex differences in mean motion parameters. We ran separate models to analyze the mean relative motion across the six runs for each participant, and to analyze the mean absolute motion across the six runs for each participant. Reported below are the main effects of run and sex and the sex × run interaction. Participants were entered as a random factor in these models.

Here we show that regardless of using mean absolute motion or mean relative motion parameters, there were no sex differences present in the data.

| **Group Descriptives** | | | | | | | | | | | | | |
| --- | --- | --- | --- | --- | --- | --- | --- | --- | --- | --- | --- | --- | --- |
|  | | **Group** | | **N** | | **Mean** | | **SD** | | **SE** | | **Coefficient of variation** | |
| Mean Absolute Motion (mm) |  | Female |  | 198 |  | 0.289 |  | 0.143 |  | 0.010 |  | 0.497 |  |
|  |  | male |  | 168 |  | 0.305 |  | 0.137 |  | 0.011 |  | 0.451 |  |
| Mean Relative Motion (mm) |  | Female |  | 198 |  | 0.093 |  | 0.034 |  | 0.002 |  | 0.360 |  |
|  |  | male |  | 168 |  | 0.103 |  | 0.031 |  | 0.002 |  | 0.300 |  |
|  | | | | | | | | | | | | | |

**Linear mixed effects model for absolute motion parameters**

| **ANOVA Summary** | | | | | | | |
| --- | --- | --- | --- | --- | --- | --- | --- |
| **Effect** | | **df** | | **F** | | **p** | |
| Run |  | 1, 59.04 |  | 2.809 |  | 0.099 |  |
| Sex |  | 1, 59.05 |  | 0.091 |  | 0.763 |  |
| Run ✻  Sex |  | 1, 59.04 |  | 0.010 |  | 0.922 |  |
|  | | | | | | | |
| *Note.*  The following variable is used as a random effects grouping factor: 'Subject'. | | | | | | | |
| *Note.*  Type III Sum of Squares | | | | | | | |

**Linear mixed effects model for relative motion parameters**

| **ANOVA Summary** | | | | | | | |
| --- | --- | --- | --- | --- | --- | --- | --- |
| **Effect** | | **df** | | **F** | | **p** | |
| Run |  | 1, 59.00 |  | 0.093 |  | 0.761 |  |
| Sex |  | 1, 59.00 |  | 0.980 |  | 0.326 |  |
| Run ✻  Sex |  | 1, 59.00 |  | 0.303 |  | 0.584 |  |
|  | | | | | | | |
| *Note.*  The following variable is used as a random effects grouping factor: 'Subject'. | | | | | | | |
| *Note.*  Type III Sum of Squares | | | | | | | |

Additional analyses in other template spaces

Between subject variability tables

| Table 3. Permutation testing of within group, between subject whole-brain voxelwise brain activation correlations  Template-Female | | | |
| --- | --- | --- | --- |
|  | **Mean Correlation ± Standard Deviation** | |  |
| Condition | **Females** | **Males** | **Independent samples t-tests** |
| Eyes | 0.300 ± 0.092 | 0.269 ± 0.072 | *t*(904) = 5.308. *p* = 1.00E-04, *d* = 0.364* |
| Jaw | 0.272 ± 0.068 | 0.230 ± .0067 | *t*(904) = 9.187, *p* = 1.00E-04, *d* = 0.619* |
| Lips | 0.271 ± 0.075 | 0.244 ± 0.074 | *t*(904) = 5.380, *p* = 1.00E-04, *d* = 0.363* |
| Tongue | 0.340 ± 0.079 | 0.327 ± 0.066 | *t*(904) = 2.634, *p* = 0.011, *d* = 0.180 |
| Upper Arms | 0.237 ± 0.089 | 0.227 ± 0.071 | *t*(904) = 1.823, *p* = 0.068, *d* = 0.125 |
| Forearms | 0.273 ± 0.081 | 0.244 ± 0.056 | *t*(904) = 6.043, *p* = 1.00E-04, *d* = 0.419* |
| Wrists | 0.342 ± 0.071 | 0.308 ± 0.069 | *t*(904) = 7.255, *p* = 1.00E-04, *d* = 0.490* |
| Fingers | 0.343 ± 0.073 | 0.326 ± 0.076 | *t*(904) = 3.405, *p* = 0.001, *d* = 0.229* |
| Left Leg | 0.249 ± 0.096 | 0.221 ± 0.101 | *t*(904) = 4.191, *p* = 2.00E-04, *d* = 0.281* |
| Right Leg | 0.229 ± 0.109 | 0.250 ± 0.100 | *t*(904) = -2.948, p = 0.004, *d* = -0.200* |
| Ankles | 0.256 ± 0.080 | 0.232 ± 0.079 | *t*(904) = 4.560, *p* = 1.00E-04, *d* = 0.308* |
| Toes | 0.271 ± 0.077 | 0.231 ± 0.065 | *t*(904) = 8.147, *p* = 1.00E-04, *d* = 0.556* |
| Note: * significant at *p* < 0.05 Bonferroni corrected [α = 0.004; (0.05/12)], two-tailed, 20,000 permutations. | | | |

| Table 3. Permutation testing of within group, between subject whole-brain voxelwise brain activation correlations  Template-Male | | | |
| --- | --- | --- | --- |
|  | **Mean Correlation ± Standard Deviation** | |  |
| Condition | **Females** | **Males** | **Independent samples t-tests** |
| Eyes | 0.299 ± 0.092 | 0.268 ± 0.073 | *t*(904) = 5.347, *p* = 1.00E-04, *d* = 0.367* |
| Jaw | 0.275 ± 0.068 | 0.233 ± 0.067 | *t*(904) = 9.282, *p* = 1.00E-04, *d* = 0.626* |
| Lips | 0.272 ± 0.076 | 0.245 ± 0.074 | *t*(904) = 5.394, *p* = 1.00E-04, *d* = 0.364* |
| Tongue | 0.344 ± 0.079 | 0.331 0.067 | *t*(904) = 2.726, *p* = 0.008, *d* = 0.186 |
| Upper Arms | 0.237 ± 0.090 | 0.227 ± 0.071 | *t*(904) = 1.767, *p* = 0.079, *d* = 0.121 |
| Forearms | 0.273 ± 0.081 | 0.244 ± 0.056 | *t*(904) = 6.017, *p* = 1.00E-04, *d* = 0.417* |
| Wrists | 0.341 ± 0.071 | 0.307 ± 0.069 | *t*(904) = 7.092, *p* = 1.00E-04, *d* = 0.479* |
| Fingers | 0.338 ± 0.073 | 0.323 ± 0.075 | *t*(904) = 3.064, *p* = 0.002, *d* = 0.206* |
| Left Leg | 0.248 ± 0.096 | 0.221 ± 0.100 | *t*(904) = 4.076, *p* = 1.00E-04, *d* = 0.274* |
| Right Leg | 0.229 ± 0.109 | 0.251 ± 0.100 | *t*(904) = -2.996, *p* = 0.002, *d* = -0.203* |
| Ankles | 0.257 ± 0.080 | 0.233 ± 0.079 | *t*(904) = 4.467, *p* = 1.00E-04, *d* = 0.301* |
| Toes | 0.272 ± 0.077 | 0.233 ± 0.065 | *t*(904) = 8.004, *p* = 1.00E-04, *d* = 0.547* |
| Note: * significant at *p* < 0.05 Bonferroni corrected [α = 0.004; (0.05/12)], two-tailed, 20,000 permutations. | | | |

| Table 3. Permutation testing of within group, between subject whole-brain voxelwise brain activation correlations  Template-Study | | | |
| --- | --- | --- | --- |
|  | **Mean Correlation ± Standard Deviation** | |  |
| Condition | **Females** | **Males** | **Independent samples t-tests** |
| Eyes | 0.299 ± 0.092 | 0.268 ± 0.072 | *t*(904) = 5.361, *p* = 1.00E-04, *d* = 0.368* |
| Jaw | 0.272 ± 0.068 | 0.230 ± 0.067 | *t*(904) = 9.214, *p* = 1.00E-04, *d* = 0.621* |
| Lips | 0.270 ± 0.075 | 0.243 ± 0.074 | *t*(904) = 5.334, *p* = 1.00E-04, *d* = 0.361* |
| Tongue | 0.341 ± 0.079 | 0.327 ± 0.066 | *t*(904) = 2.791 *p* = 0.005, *d* = 0.191 |
| Upper Arms | 0.236 ± 0.089 | 0.226 ± 0.071 | *t*(904) = 1.754, *p* = 0.081, *d* = 0.120 |
| Forearms | 0.272 ± 0.081 | 0.243 ± 0.056 | *t*(904) = 5.927, *p* = 1.00E-04, *d* = 0.411* |
| Wrists | 0.340 ± 0.071 | 0.306 ± 0.069 | *t*(904) = 7.107, *p* = 1.00E-04, *d* = 0.480* |
| Fingers | 0.339 ± 0.073 | 0.323 ± 0.075 | *t*(904) = 3.212, *p* = 0.001, *d* = 0.216* |
| Left Leg | 0.247 ± 0.096 | 0.220 ± 0.100 | *t*(904) = 4.119, *p* = 1.00E-04, *d* = 0.277* |
| Right Leg | 0.228 ± 0.109 | 0.249 ± 0.099 | *t*(904) = -2.997, *p* = 0.003, *d* = -0.203* |
| Ankles | 0.256 ± 0.080 | 0.232 ± 0.079 | *t*(904) = 4.508, *p* = 1.00E-04, *d* = 0.304* |
| Toes | 0.271 ± 0.077 | 0.232 ± 0.065 | *t*(904) = 8.050, *p* = 1.00E-04, *d* = 0.550* |
| Note: * significant at *p* < 0.05 Bonferroni corrected [α = 0.004; (0.05/12)], two-tailed, 20,000 permutations. | | | |

Similarity between significant clusters tables

| **Table 2. Group-level whole brain spatial maps and correlations between sexes** | | | | |
| --- | --- | --- | --- | --- |
| **template-female** | | | | |
|  | **Total number of significant voxels*** | | **Spatial map similarity** | |
| **Condition** | **Females** | **Males** | **Difference (%)**** | ***r*-values***** |
| **Eyes** | 35,754 | 48,801 | 36.49 | 0.69 |
| **Jaw** | 15,691 | 35,469 | 126.05 | 0.6 |
| **Lips** | 25,911 | 36,800 | 42.02 | 0.67 |
| **Tongue** | 30,722 | 45,363 | 47.66 | 0.68 |
| **Upper Arms** | 8,552 | 31,325 | 266.29 | 0.47 |
| **Forearms** | 25,170 | 43,997 | 74.80 | 0.62 |
| **Wrists** | 26,393 | 50,968 | 93.11 | 0.65 |
| **Fingers** | 28,028 | 45,558 | 62.54 | 0.67 |
| **Left Leg** | 5,619 | 9,206 | 63.84 | 0.69 |
| **Right Leg** | 4,670 | 11,482 | 145.87 | 0.61 |
| **Ankles** | 11,335 | 34,954 | 208.37 | 0.51 |
| **Toes** | 30,751 | 39,137 | 27.27 | 0.69 |
| *Voxel size = 2 × 2 × 2 mm | | | | |
| **Percent difference of voxels activated in each condition for males compared to females | | | | |
| *** Pearson’s *r* correlation coefficients from a cross-correlation analyses between females and males | | | | |

| **Table 2. Group-level whole brain spatial maps and correlations between sexes** | | | | |
| --- | --- | --- | --- | --- |
| **template-male** | | | | |
|  | **Total number of significant voxels*** | | **Spatial map similarity** | |
| **Condition** | **Females** | **Males** | **Difference (%)**** | ***r*-values***** |
| **Eyes** | 39,419 | 52,864 | 34.11 | 0.69 |
| **Jaw** | 17,501 | 35,219 | 101.24 | 0.63 |
| **Lips** | 30,340 | 38,794 | 27.86 | 0.69 |
| **Tongue** | 34,953 | 48,569 | 38.96 | 0.69 |
| **Upper Arms** | 9,406 | 35,108 | 273.25 | 0.47 |
| **Forearms** | 27,387 | 49,059 | 79.13 | 0.62 |
| **Wrists** | 30,034 | 54,396 | 81.11 | 0.65 |
| **Fingers** | 30,987 | 50,361 | 62.52 | 0.67 |
| **Left Leg** | 6,321 | 10,043 | 58.88 | 0.7 |
| **Right Leg** | 5,154 | 12,777 | 147.90 | 0.6 |
| **Ankles** | 11,547 | 40,485 | 250.61 | 0.48 |
| **Toes** | 35,909 | 43,901 | 22.26 | 0.69 |
| *Voxel size = 2 × 2 × 2 mm | | | | |
| **Percent difference of voxels activated in each condition for males compared to females | | | | |
| *** Pearson’s *r* correlation coefficients from a cross-correlation analyses between females and males | | | | |

| **Table 2. Group-level whole brain spatial maps and correlations between sexes** | | | | |
| --- | --- | --- | --- | --- |
| **template-study** | | | | |
|  | **Total number of significant voxels*** | | **Spatial map similarity** | |
| **Condition** | **Females** | **Males** | **Difference (%)**** | ***r*-values***** |
| **Eyes** | 36,037 | 48,494 | 34.57 | 0.7 |
| **Jaw** | 16,722 | 32,603 | 94.97 | 0.64 |
| **Lips** | 27,268 | 37,817 | 38.69 | 0.68 |
| **Tongue** | 32,380 | 44,402 | 37.13 | 0.68 |
| **Upper Arms** | 8,708 | 31,984 | 267.29 | 0.47 |
| **Forearms** | 24,374 | 43,957 | 80.34 | 0.61 |
| **Wrists** | 27,255 | 51,707 | 89.72 | 0.65 |
| **Fingers** | 29,575 | 47,360 | 60.14 | 0.67 |
| **Left Leg** | 5,682 | 9,490 | 67.02 | 0.69 |
| **Right Leg** | 4,716 | 11,481 | 143.45 | 0.61 |
| **Ankles** | 12,823 | 36,383 | 183.73 | 0.52 |
| **Toes** | 31,735 | 40,486 | 27.58 | 0.69 |
| *Voxel size = 2 × 2 × 2 mm | | | | |
| **Percent difference of voxels activated in each condition for males compared to females | | | | |
| *** Pearson’s *r* correlation coefficients from a cross-correlation analyses between females and males | | | | |

Figures and table show significant differences with templates likely caused by the male template resulting in slightly more voxels for both sexes, but there was no template × sex interation. These data show that regardless of task (each dot and line represents one of the motor tasks) changing the template space did not significantly change the difference between females and males in the number of significant voxels across the brain.


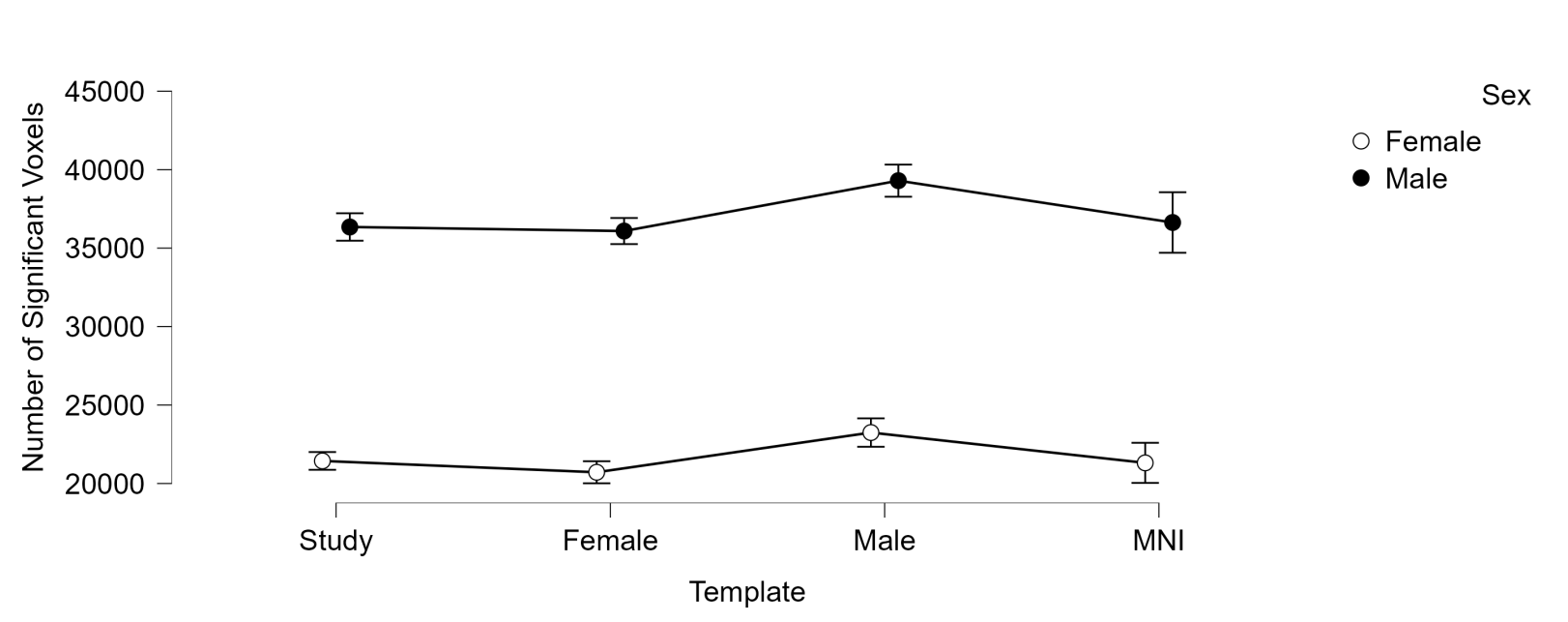


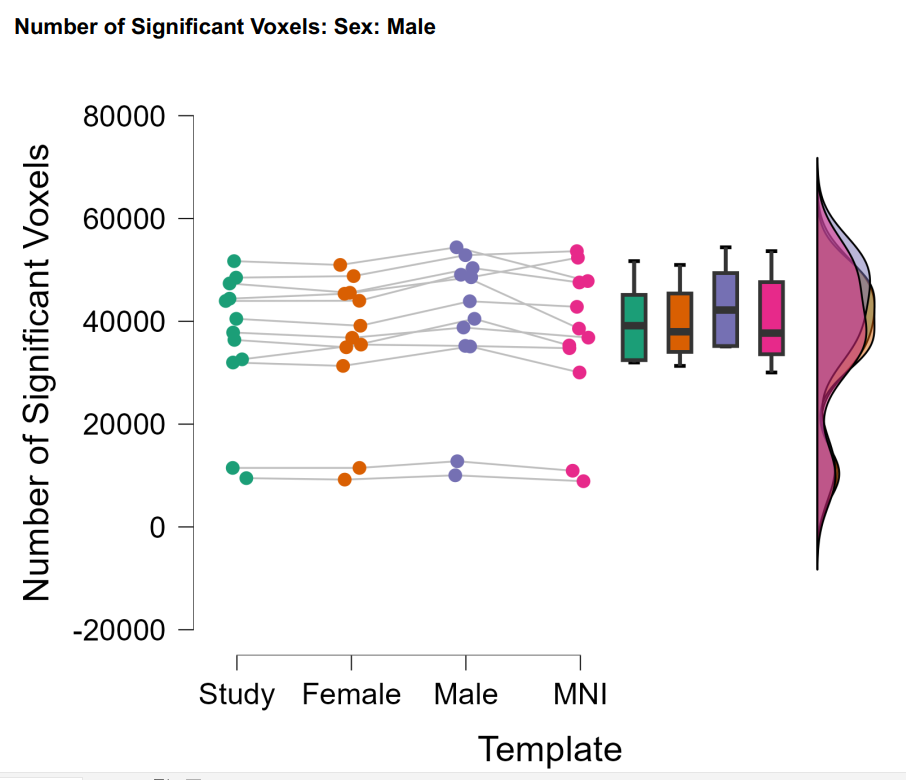

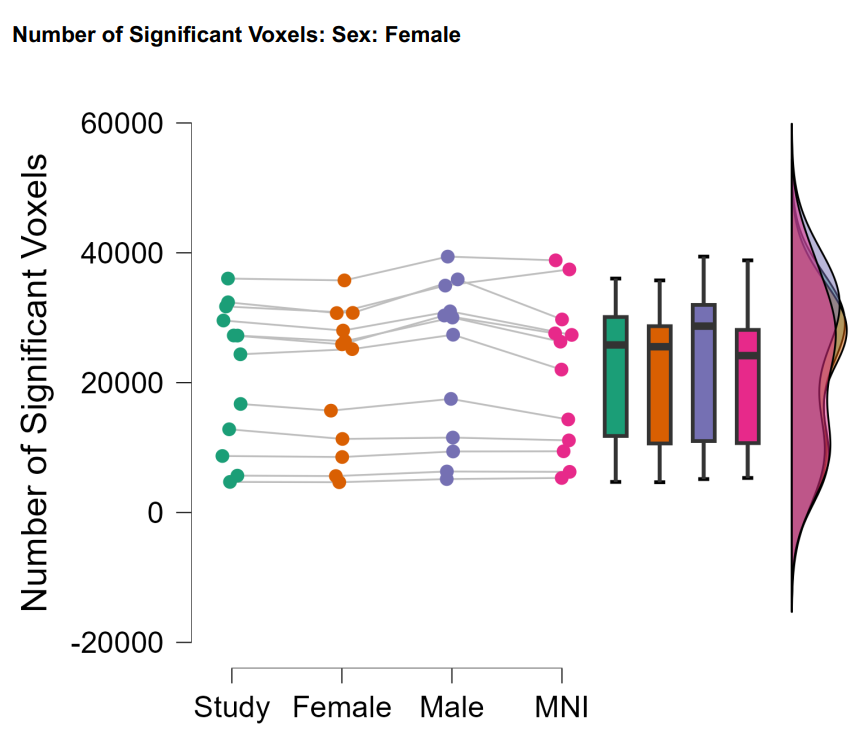


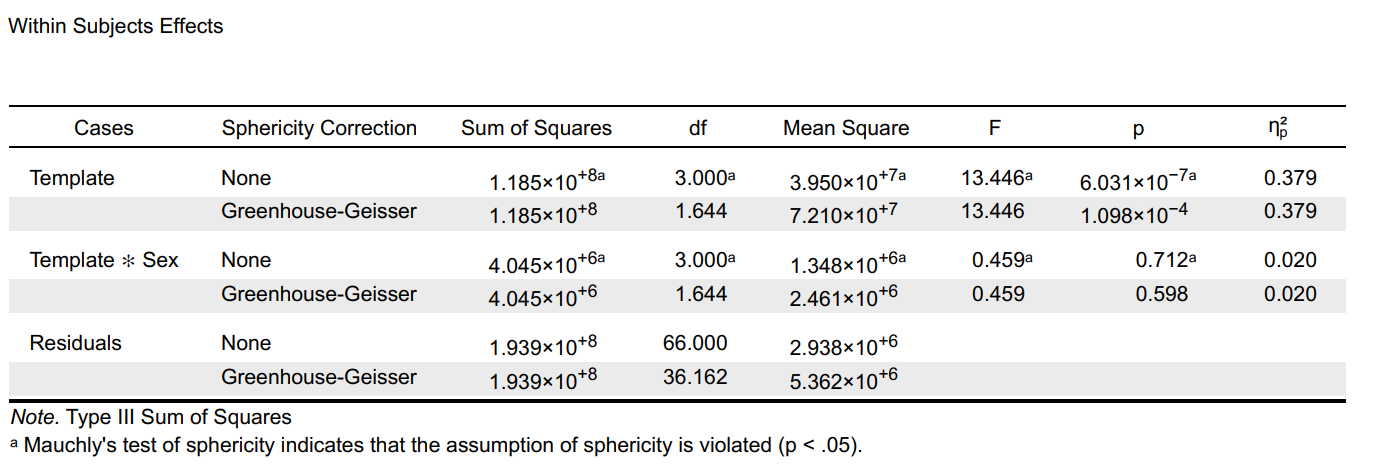


Sex contrast tables

Ankles

| **Males > Females during ankle movements in the female template space** | | | | | | | |
| --- | --- | --- | --- | --- | --- | --- | --- |
| Voxels | MAX | MAX X (vox) | MAX Y (vox) | MAX Z (vox) | COG X (vox) | COG Y (vox) | COG Z (vox) |
| 1039 | 5.59 | 34 | 64 | 79 | 39.6 | 60.1 | 88.7 |
| 707 | 4.35 | 68 | 29 | 81 | 62.8 | 28.3 | 83.7 |
| 304 | 4.45 | 59 | 45 | 87 | 61.3 | 40 | 90.6 |
| 214 | 4.27 | 72 | 33 | 99 | 73.9 | 33.1 | 96.3 |
| 201 | 4 | 46 | 37 | 90 | 42.7 | 38.7 | 89.2 |
| 94 | 4.29 | 90 | 41 | 90 | 89.5 | 39.8 | 92.2 |
| 32 | 4.5 | 44 | 37 | 98 | 42.7 | 37.3 | 97.7 |
| 31 | 5.09 | 62 | 22 | 82 | 61.9 | 22 | 83.2 |
| 27 | 3.54 | 39 | 60 | 79 | 41.2 | 58.4 | 79.5 |
| 21 | 4.78 | 45 | 49 | 78 | 45.2 | 49.9 | 77.8 |
| 17 | 3.33 | 48 | 48 | 101 | 48.9 | 48.2 | 102 |
| 16 | 3.52 | 49 | 56 | 92 | 48.6 | 56.4 | 92.5 |
| 16 | 3.82 | 53 | 37 | 80 | 52.9 | 37.5 | 78.4 |
| 14 | 4.18 | 95 | 52 | 87 | 95.4 | 51.4 | 87.1 |
| 13 | 4.02 | 36 | 50 | 80 | 35.7 | 51.1 | 80.1 |
| 9 | 3.43 | 35 | 40 | 92 | 35.6 | 39.7 | 92.8 |
| 8 | 3.2 | 58 | 26 | 90 | 57.6 | 25.8 | 89.1 |
| 7 | 4.55 | 94 | 64 | 77 | 93.7 | 63.7 | 77.6 |
| 7 | 3.15 | 69 | 39 | 97 | 68.4 | 39.2 | 97.1 |
| 6 | 2.84 | 76 | 28 | 87 | 76.3 | 26.4 | 86.5 |
| 6 | 3.82 | 46 | 64 | 78 | 46.6 | 63.2 | 78.6 |
| 5 | 3.47 | 40 | 41 | 99 | 39.8 | 41 | 98.6 |
| 4 | 3.13 | 53 | 60 | 92 | 53.2 | 59.7 | 92.3 |
| 4 | 3.86 | 42 | 49 | 81 | 41.3 | 48.3 | 81 |
| 4 | 3.73 | 60 | 32 | 79 | 60.2 | 32.2 | 79.2 |
| 3 | 3.34 | 36 | 38 | 90 | 36.6 | 38.9 | 89.7 |
| 2 | 3.81 | 32 | 55 | 85 | 32.5 | 55 | 85 |
| 2 | 3.26 | 45 | 57 | 92 | 44.5 | 57 | 92 |
| 2 | 3.23 | 46 | 62 | 95 | 46.5 | 62 | 95 |
| 1 | 3.49 | 69 | 40 | 89 | 69 | 40 | 89 |
| 1 | 3.02 | 73 | 29 | 87 | 73 | 29 | 87 |
| 1 | 4.69 | 35 | 78 | 81 | 35 | 78 | 81 |

| **Males > Females during ankle movements in the male template space** | | | | | | | |
| --- | --- | --- | --- | --- | --- | --- | --- |
| Voxels | MAX | MAX X (vox) | MAX Y (vox) | MAX Z (vox) | COG X (vox) | COG Y (vox) | COG Z (vox) |
| 932 | 5.31 | 63 | 21 | 75 | 63.2 | 26.9 | 76.8 |
| 624 | 4.41 | 76 | 30 | 91 | 67.2 | 35.8 | 86.6 |
| 500 | 5.13 | 35 | 68 | 74 | 33 | 67 | 77.6 |
| 470 | 4.35 | 43 | 52 | 93 | 44.8 | 54.9 | 89.6 |
| 189 | 4.41 | 89 | 36 | 88 | 89.9 | 37.9 | 86.6 |
| 52 | 3.89 | 45 | 36 | 82 | 43.2 | 40.9 | 81.7 |
| 50 | 3.65 | 46 | 69 | 81 | 44.9 | 67.2 | 82.4 |
| 25 | 4.14 | 96 | 51 | 84 | 96.8 | 51.2 | 83.9 |
| 25 | 4.72 | 43 | 35 | 90 | 42.3 | 35.8 | 89.7 |
| 23 | 3.05 | 54 | 61 | 87 | 53.1 | 61.1 | 87.2 |
| 22 | 3.65 | 40 | 62 | 74 | 40.8 | 61 | 74.2 |
| 20 | 4.5 | 94 | 66 | 76 | 95.4 | 66.4 | 74.8 |
| 16 | 3.37 | 48 | 47 | 95 | 48.4 | 47.2 | 95.3 |
| 15 | 3.64 | 54 | 48 | 94 | 54.3 | 48 | 94.1 |
| 11 | 3.32 | 69 | 38 | 90 | 68.4 | 37.6 | 90.1 |
| 9 | 4.33 | 45 | 50 | 72 | 45 | 50.6 | 71.8 |
| 5 | 3.74 | 40 | 73 | 80 | 40.8 | 73 | 79.6 |
| 4 | 3.05 | 81 | 26 | 85 | 80.5 | 25.5 | 85 |
| 4 | 3.51 | 55 | 48 | 91 | 55 | 48.3 | 91 |
| 3 | 4.32 | 27 | 62 | 71 | 27.7 | 61.7 | 71 |
| 2 | 3.3 | 42 | 35 | 84 | 42.5 | 35 | 84 |
| 2 | 3.73 | 32 | 56 | 79 | 32.5 | 56 | 79 |
| 2 | 4.59 | 84 | 65 | 96 | 83.5 | 65 | 95.5 |
| 1 | 2.98 | 41 | 67 | 84 | 41 | 67 | 84 |
| 1 | 3.27 | 59 | 25 | 83 | 59 | 25 | 83 |
| 1 | 3.22 | 88 | 34 | 82 | 88 | 34 | 82 |
| 1 | 4.35 | 82 | 63 | 96 | 82 | 63 | 96 |
| 1 | 3.66 | 45 | 72 | 77 | 45 | 72 | 77 |
| 1 | 3.76 | 42 | 50 | 75 | 42 | 50 | 75 |
| 1 | 4.21 | 53 | 37 | 73 | 53 | 37 | 73 |

| **Males > Females during ankle movements in the study template space** | | | | | | | |
| --- | --- | --- | --- | --- | --- | --- | --- |
| Voxels | MAX | MAX X (vox) | MAX Y (vox) | MAX Z (vox) | COG X (vox) | COG Y (vox) | COG Z (vox) |
| 1479 | 5.31 | 62 | 22 | 82 | 63.6 | 31.7 | 87.2 |
| 1284 | 5.14 | 35 | 64 | 79 | 40.9 | 59.4 | 89.3 |
| 239 | 4.03 | 46 | 37 | 90 | 41.3 | 38.5 | 89.5 |
| 235 | 4.2 | 90 | 41 | 90 | 88.7 | 38.8 | 91.4 |
| 60 | 4.26 | 95 | 51 | 87 | 95.2 | 51.2 | 87.1 |
| 34 | 4.64 | 94 | 64 | 77 | 94.1 | 64.3 | 77.1 |
| 25 | 4.93 | 45 | 49 | 78 | 45 | 49.9 | 77.5 |
| 22 | 4.38 | 44 | 37 | 98 | 42.8 | 37.4 | 97.6 |
| 22 | 3.07 | 37 | 71 | 80 | 36.7 | 70.6 | 81.5 |
| 14 | 3.81 | 46 | 64 | 78 | 46.6 | 63.2 | 79 |
| 13 | 3.9 | 36 | 50 | 80 | 35.7 | 51.3 | 80.1 |
| 11 | 3.14 | 69 | 39 | 97 | 68 | 39.1 | 97.2 |
| 10 | 3.89 | 60 | 32 | 79 | 59.8 | 31.7 | 78.5 |
| 8 | 3.77 | 33 | 55 | 85 | 32.2 | 55.2 | 85.2 |
| 7 | 3.87 | 42 | 49 | 81 | 41.5 | 48.2 | 81 |
| 7 | 3.34 | 96 | 44 | 86 | 95.7 | 44.3 | 85.7 |
| 4 | 3.21 | 38 | 52 | 91 | 38.4 | 51.8 | 91.2 |
| 3 | 2.92 | 67 | 37 | 91 | 67.3 | 37 | 91.3 |
| 2 | 2.51 | 44 | 49 | 92 | 43.5 | 49 | 92 |
| 2 | 3.75 | 98 | 60 | 77 | 97.5 | 60 | 77 |
| 2 | 3.29 | 29 | 55 | 83 | 29.5 | 55 | 83 |
| 2 | 3.75 | 93 | 59 | 75 | 93.5 | 59 | 75 |
| 1 | 2.3 | 49 | 60 | 103 | 49 | 60 | 103 |
| 1 | 3.53 | 58 | 26 | 90 | 58 | 26 | 90 |
| 1 | 3.22 | 95 | 49 | 82 | 95 | 49 | 82 |
| 1 | 3.45 | 39 | 55 | 78 | 39 | 55 | 78 |
| 1 | 2.92 | 73 | 36 | 75 | 73 | 36 | 75 |
| 1 | 3.75 | 95 | 62 | 74 | 95 | 62 | 74 |
| 1 | 4.67 | 83 | 64 | 99 | 83 | 64 | 99 |

Eyes

None

Fingers

| **Males > Females during finger movements in the female template space** | | | | | | | |
| --- | --- | --- | --- | --- | --- | --- | --- |
| Voxels | MAX | MAX X (vox) | MAX Y (vox) | MAX Z (vox) | COG X (vox) | COG Y (vox) | COG Z (vox) |
| 3271 | 5.01 | 41 | 38 | 81 | 60.3 | 33.7 | 82.2 |
| 1589 | 4.91 | 33 | 64 | 79 | 40.5 | 57.8 | 80.8 |
| 1058 | 5.08 | 70 | 49 | 95 | 62.6 | 47.1 | 98.4 |
| 228 | 4.08 | 58 | 56 | 90 | 62.5 | 57 | 88.7 |
| 165 | 4.09 | 86 | 38 | 82 | 85.1 | 35.6 | 79.1 |
| 97 | 4.75 | 53 | 31 | 52 | 50.2 | 28.9 | 53.5 |
| 51 | 4.07 | 63 | 89 | 77 | 63.6 | 87.9 | 78 |
| 49 | 4.08 | 44 | 36 | 98 | 42.8 | 37.4 | 97.9 |
| 40 | 4.91 | 63 | 76 | 81 | 63.1 | 77.7 | 80.8 |
| 36 | 4.58 | 81 | 53 | 79 | 82.2 | 53.1 | 78.4 |
| 26 | 4.07 | 98 | 61 | 76 | 95.2 | 63.7 | 77.6 |
| 21 | 3.76 | 38 | 36 | 75 | 39.1 | 36.4 | 74.4 |
| 14 | 3.2 | 30 | 55 | 85 | 31.2 | 54.9 | 84.6 |
| 11 | 4.16 | 38 | 49 | 94 | 37.6 | 48.6 | 94.8 |
| 10 | 3.09 | 48 | 49 | 81 | 47.4 | 49.4 | 82 |
| 9 | 4.15 | 81 | 64 | 76 | 80.7 | 64.5 | 75.6 |
| 8 | 3.29 | 50 | 49 | 90 | 50.4 | 49.4 | 90.2 |
| 8 | 3.53 | 97 | 61 | 79 | 97.6 | 60.9 | 79.1 |
| 7 | 3.48 | 51 | 46 | 78 | 50.7 | 46.9 | 78.2 |
| 6 | 3.87 | 75 | 44 | 88 | 75.3 | 45.4 | 86.9 |
| 6 | 4.16 | 46 | 77 | 78 | 45.5 | 77.3 | 77.7 |
| 5 | 2.64 | 49 | 25 | 90 | 48.8 | 25.6 | 90.4 |
| 5 | 4.89 | 67 | 88 | 73 | 66.8 | 87.6 | 73 |
| 5 | 2.91 | 43 | 67 | 75 | 42.4 | 67 | 75.4 |
| 4 | 3.3 | 63 | 92 | 78 | 63.5 | 93 | 78 |
| 4 | 3.54 | 39 | 74 | 80 | 38.3 | 74.7 | 80 |
| 4 | 4.14 | 74 | 52 | 81 | 74.2 | 50.8 | 81 |
| 4 | 3.99 | 84 | 55 | 71 | 83.5 | 54.3 | 71.2 |
| 3 | 3.53 | 52 | 47 | 85 | 51.7 | 47 | 85.3 |
| 3 | 3.83 | 67 | 83 | 84 | 67 | 82.7 | 84.6 |
| 3 | 3.51 | 50 | 35 | 101 | 50.3 | 35 | 101 |
| 2 | 3.08 | 49 | 48 | 85 | 49 | 48 | 84.5 |
| 2 | 4 | 79 | 55 | 83 | 78.5 | 55 | 83 |
| 2 | 3.6 | 79 | 48 | 84 | 79.5 | 48 | 83.5 |
| 2 | 2.43 | 74 | 27 | 88 | 74 | 26.5 | 88 |
| 2 | 4.52 | 80 | 60 | 78 | 80.5 | 60 | 77.5 |
| 2 | 3.14 | 68 | 57 | 90 | 68 | 56.5 | 90 |
| 2 | 3.87 | 45 | 45 | 92 | 45 | 45.5 | 91.5 |
| 1 | 3.87 | 93 | 60 | 84 | 93 | 60 | 84 |
| 1 | 3.52 | 74 | 42 | 84 | 74 | 42 | 84 |
| 1 | 3.98 | 81 | 53 | 73 | 81 | 53 | 73 |
| 1 | 3.27 | 60 | 92 | 81 | 60 | 92 | 81 |
| 1 | 3.64 | 45 | 46 | 76 | 45 | 46 | 76 |
| 1 | 3.61 | 71 | 56 | 92 | 71 | 56 | 92 |
| 1 | 2.99 | 85 | 40 | 76 | 85 | 40 | 76 |
| 1 | 3.38 | 41 | 60 | 74 | 41 | 60 | 74 |

| **Males > Females during finger movements in the male template space** | | | | | | | |
| --- | --- | --- | --- | --- | --- | --- | --- |
| Voxels | MAX | MAX X (vox) | MAX Y (vox) | MAX Z (vox) | COG X (vox) | COG Y (vox) | COG Z (vox) |
| 5441 | 5.12 | 62 | 40 | 82 | 61.1 | 38 | 79.5 |
| 1839 | 4.95 | 45 | 72 | 77 | 39.6 | 59.7 | 75.3 |
| 111 | 4.54 | 82 | 54 | 75 | 83.5 | 54.2 | 74.4 |
| 111 | 4.15 | 84 | 31 | 71 | 87.1 | 34.7 | 73.5 |
| 63 | 3.88 | 81 | 67 | 74 | 81.2 | 67.9 | 73.4 |
| 49 | 3.97 | 43 | 35 | 90 | 41.5 | 36.9 | 89.9 |
| 47 | 3.75 | 52 | 48 | 79 | 48.7 | 48.5 | 77.4 |
| 38 | 4.04 | 85 | 57 | 67 | 83.1 | 55.2 | 68.4 |
| 32 | 4.65 | 81 | 62 | 75 | 81.4 | 62.1 | 74.5 |
| 22 | 3.85 | 79 | 60 | 67 | 78.4 | 57.1 | 71.2 |
| 22 | 4.09 | 99 | 63 | 74 | 96.9 | 65.5 | 74.7 |
| 20 | 4.2 | 37 | 48 | 88 | 37.1 | 48 | 88 |
| 16 | 3.79 | 45 | 45 | 85 | 44.9 | 44.7 | 85 |
| 14 | 3.54 | 52 | 35 | 88 | 51.7 | 34.9 | 88.6 |
| 12 | 3.35 | 46 | 32 | 92 | 48.2 | 32.8 | 92.4 |
| 11 | 3.29 | 52 | 48 | 72 | 50.8 | 48.5 | 72.3 |
| 6 | 4.6 | 63 | 81 | 78 | 63.5 | 79.7 | 78.3 |
| 6 | 3.03 | 79 | 60 | 79 | 80.1 | 59.7 | 79.6 |
| 6 | 4.37 | 56 | 27 | 48 | 56.2 | 27.5 | 47.8 |
| 5 | 3.36 | 50 | 50 | 84 | 50 | 49.8 | 84 |
| 5 | 3.89 | 99 | 63 | 77 | 98.8 | 62.4 | 76.6 |
| 5 | 3.71 | 64 | 94 | 75 | 63.4 | 94.6 | 74.8 |
| 4 | 3.23 | 35 | 65 | 87 | 35.2 | 64.8 | 87 |
| 3 | 2.78 | 47 | 77 | 76 | 47.3 | 76.7 | 76.3 |
| 3 | 3.81 | 64 | 92 | 75 | 64.3 | 91.7 | 74.7 |
| 3 | 3.4 | 55 | 47 | 60 | 54.4 | 47 | 59.7 |
| 3 | 3.22 | 27 | 50 | 67 | 27 | 50.3 | 66.7 |
| 3 | 2.86 | 74 | 44 | 88 | 74.6 | 44.3 | 88 |
| 3 | 3.58 | 77 | 63 | 70 | 77.3 | 63 | 69.7 |
| 3 | 3.47 | 49 | 40 | 60 | 49.3 | 40.3 | 60 |
| 2 | 3.99 | 46 | 33 | 48 | 46 | 33 | 48.5 |
| 2 | 4.08 | 54 | 67 | 73 | 54 | 66.5 | 73 |
| 2 | 3.46 | 48 | 51 | 60 | 48.5 | 50.5 | 60 |
| 2 | 2.96 | 92 | 56 | 77 | 92.5 | 56 | 77 |
| 2 | 3.4 | 44 | 40 | 86 | 44.5 | 40.5 | 86 |
| 2 | 4.32 | 52 | 60 | 78 | 51.5 | 60 | 78.5 |
| 2 | 3.09 | 53 | 39 | 60 | 52.5 | 39 | 60 |
| 1 | 3.37 | 39 | 49 | 83 | 39 | 49 | 83 |
| 1 | 3.02 | 63 | 58 | 80 | 63 | 58 | 80 |
| 1 | 3.94 | 65 | 97 | 78 | 65 | 97 | 78 |
| 1 | 3.4 | 55 | 44 | 60 | 55 | 44 | 60 |
| 1 | 3.44 | 79 | 69 | 77 | 79 | 69 | 77 |
| 1 | 3.64 | 47 | 42 | 61 | 47 | 42 | 61 |
| 1 | 2.94 | 48 | 47 | 74 | 48 | 47 | 74 |
| 1 | 3.64 | 85 | 38 | 73 | 85 | 38 | 73 |
| 1 | 3.62 | 80 | 64 | 67 | 80 | 64 | 67 |
| 1 | 3.22 | 76 | 60 | 69 | 76 | 60 | 69 |
| 1 | 3.98 | 53 | 28 | 47 | 53 | 28 | 47 |

| **Males > Females during finger movements in the study template space** | | | | | | | |
| --- | --- | --- | --- | --- | --- | --- | --- |
| Voxels | MAX | MAX X (vox) | MAX Y (vox) | MAX Z (vox) | COG X (vox) | COG Y (vox) | COG Z (vox) |
| 3458 | 4.94 | 40 | 39 | 81 | 60.1 | 33.8 | 82 |
| 1559 | 4.93 | 34 | 64 | 79 | 40.1 | 57 | 79.8 |
| 1371 | 5.29 | 70 | 49 | 95 | 62.6 | 48.9 | 96.5 |
| 395 | 4.85 | 67 | 88 | 73 | 64.1 | 85.5 | 79.4 |
| 340 | 4.2 | 82 | 32 | 77 | 84.9 | 35 | 78.8 |
| 97 | 4.49 | 55 | 25 | 54 | 49.4 | 28.6 | 53.8 |
| 91 | 4.04 | 43 | 37 | 98 | 43.9 | 37.3 | 98.4 |
| 66 | 3.57 | 57 | 87 | 73 | 62.9 | 88.7 | 70.2 |
| 64 | 4.58 | 81 | 53 | 79 | 82.2 | 53 | 78.3 |
| 48 | 3.62 | 39 | 62 | 98 | 41.6 | 59.7 | 98.5 |
| 35 | 4.08 | 81 | 64 | 76 | 80.6 | 65.2 | 76 |
| 31 | 3.82 | 45 | 46 | 91 | 44.7 | 44.7 | 92.2 |
| 29 | 5.04 | 53 | 31 | 52 | 54.2 | 30.9 | 51.2 |
| 22 | 3.22 | 29 | 56 | 84 | 31.3 | 54.8 | 84.5 |
| 21 | 3.92 | 98 | 61 | 76 | 95.1 | 63.6 | 77.4 |
| 20 | 4.29 | 84 | 55 | 71 | 81.9 | 53.2 | 71.7 |
| 12 | 4.98 | 80 | 60 | 78 | 80.7 | 60.3 | 77.8 |
| 9 | 3.98 | 60 | 74 | 74 | 59.2 | 74.1 | 73.5 |
| 9 | 3.45 | 40 | 36 | 55 | 39.3 | 36.2 | 55 |
| 5 | 3.3 | 36 | 63 | 93 | 36.4 | 63.4 | 92.6 |
| 5 | 3.81 | 57 | 76 | 78 | 56 | 76.4 | 77.8 |
| 4 | 3.18 | 41 | 51 | 72 | 40.8 | 49.8 | 71.3 |
| 4 | 4.62 | 65 | 78 | 69 | 64.5 | 78 | 68.7 |
| 4 | 3.62 | 97 | 61 | 79 | 97.5 | 61 | 79.5 |
| 3 | 4.19 | 38 | 49 | 94 | 37.7 | 48.4 | 94.6 |
| 3 | 4.16 | 74 | 52 | 81 | 74 | 51 | 81 |
| 3 | 3.13 | 48 | 49 | 81 | 47 | 49.3 | 82 |
| 3 | 3.36 | 70 | 88 | 81 | 69.7 | 88 | 80.7 |
| 3 | 4.15 | 66 | 81 | 66 | 65.7 | 81.3 | 66 |
| 3 | 3.49 | 89 | 42 | 78 | 88.7 | 41.4 | 78 |
| 2 | 3.42 | 50 | 48 | 79 | 50 | 48 | 78.5 |
| 2 | 3.39 | 48 | 38 | 94 | 47.5 | 38 | 94 |
| 2 | 2.75 | 75 | 38 | 72 | 75 | 38 | 71.5 |
| 2 | 2.53 | 80 | 41 | 78 | 80.5 | 41 | 78 |
| 2 | 4.27 | 70 | 84 | 89 | 70 | 83.5 | 89 |
| 1 | 4.34 | 52 | 30 | 97 | 52 | 30 | 97 |
| 1 | 2.93 | 60 | 31 | 51 | 60 | 31 | 51 |
| 1 | 2.83 | 43 | 61 | 95 | 43 | 61 | 95 |
| 1 | 3.45 | 63 | 71 | 70 | 63 | 71 | 70 |
| 1 | 2.95 | 34 | 68 | 90 | 34 | 68 | 90 |
| 1 | 3.55 | 63 | 76 | 71 | 63 | 76 | 71 |
| 1 | 4.1 | 75 | 44 | 88 | 75 | 44 | 88 |
| 1 | 3.04 | 69 | 57 | 86 | 69 | 57 | 86 |
| 1 | 3.3 | 64 | 70 | 72 | 64 | 70 | 72 |
| 1 | 4.25 | 52 | 58 | 83 | 52 | 58 | 83 |
| 1 | 3.46 | 64 | 74 | 73 | 64 | 74 | 73 |
| 1 | 3.44 | 41 | 60 | 74 | 41 | 60 | 74 |
| 1 | 3.1 | 43 | 74 | 76 | 43 | 74 | 76 |
| 1 | 4.32 | 44 | 76 | 80 | 44 | 76 | 80 |
| 1 | 3.67 | 38 | 76 | 80 | 38 | 76 | 80 |
| 1 | 3.78 | 39 | 74 | 80 | 39 | 74 | 80 |
| 1 | 4.31 | 79 | 75 | 79 | 79 | 75 | 79 |
| 1 | 3.01 | 76 | 45 | 97 | 76 | 45 | 97 |
| 1 | 3.49 | 51 | 46 | 78 | 51 | 46 | 78 |
| 1 | 3.27 | 84 | 43 | 78 | 84 | 43 | 78 |
| 1 | 3.66 | 52 | 47 | 85 | 52 | 47 | 85 |

Forearms

| **Males > Females during forearm movements in the female template space** | | | | | | | |
| --- | --- | --- | --- | --- | --- | --- | --- |
| Voxels | MAX | MAX X (vox) | MAX Y (vox) | MAX Z (vox) | COG X (vox) | COG Y (vox) | COG Z (vox) |
| 29 | 5.32 | 62 | 22 | 82 | 61.8 | 21.2 | 81.3 |
| 7 | 5.58 | 34 | 63 | 79 | 34 | 63.4 | 79.1 |
| 4 | 5.59 | 63 | 65 | 78 | 63 | 65.5 | 78 |

| **Males > Females during forearm movements in the male template space** | | | | | | | |
| --- | --- | --- | --- | --- | --- | --- | --- |
| Voxels | MAX | MAX X (vox) | MAX Y (vox) | MAX Z (vox) | COG X (vox) | COG Y (vox) | COG Z (vox) |
| 40 | 5.34 | 63 | 21 | 75 | 62.6 | 20.2 | 73.7 |
| 19 | 5.65 | 34 | 67 | 74 | 34.4 | 67.1 | 73.9 |
| 5 | 5.67 | 63 | 68 | 74 | 63.2 | 68.2 | 74 |
| 3 | 3.78 | 65 | 24 | 78 | 65 | 23.3 | 79 |
| 2 | 4.19 | 34 | 66 | 79 | 34 | 66.5 | 79 |

| **Males > Females during forearm movements in the study template space** | | | | | | | |
| --- | --- | --- | --- | --- | --- | --- | --- |
| Voxels | MAX | MAX X (vox) | MAX Y (vox) | MAX Z (vox) | COG X (vox) | COG Y (vox) | COG Z (vox) |
| 34 | 5.59 | 62 | 22 | 82 | 61.8 | 21.5 | 81.3 |
| 7 | 5.39 | 34 | 64 | 79 | 34.3 | 63.7 | 79.3 |
| 6 | 5.79 | 63 | 65 | 78 | 62.5 | 65.5 | 78 |
| 2 | 3.9 | 68 | 29 | 80 | 68.5 | 29.5 | 80 |
| 2 | 3.85 | 67 | 26 | 81 | 67 | 25.5 | 81 |
| 2 | 3.54 | 61 | 24 | 87 | 61.5 | 24 | 87 |
| 1 | 4.19 | 55 | 34 | 79 | 55 | 34 | 79 |
| 1 | 4.26 | 55 | 30 | 83 | 55 | 30 | 83 |

Jaw

| **Males > Females during jaw movements in the female template space** | | | | | | | |
| --- | --- | --- | --- | --- | --- | --- | --- |
| Voxels | MAX | MAX X (vox) | MAX Y (vox) | MAX Z (vox) | COG X (vox) | COG Y (vox) | COG Z (vox) |
| 4285 | 4.75 | 42 | 39 | 97 | 58.6 | 42.8 | 92.2 |
| 142 | 3.99 | 40 | 61 | 99 | 43.2 | 61.5 | 97.3 |
| 132 | 4.14 | 44 | 39 | 77 | 41.2 | 35 | 76.8 |
| 28 | 4.45 | 72 | 27 | 86 | 72.7 | 27.3 | 85.1 |
| 28 | 4.4 | 63 | 22 | 82 | 62.9 | 22.7 | 83.5 |
| 28 | 3.91 | 69 | 33 | 79 | 69.4 | 32.9 | 79.9 |
| 20 | 3.81 | 73 | 38 | 75 | 71.9 | 37 | 76.1 |
| 19 | 3.41 | 35 | 35 | 73 | 34.4 | 34.9 | 72.4 |
| 11 | 3.69 | 58 | 36 | 101 | 56.9 | 35.6 | 102 |
| 9 | 3.31 | 42 | 69 | 92 | 41.8 | 68.7 | 92.6 |
| 6 | 3.52 | 44 | 58 | 77 | 44 | 57.8 | 77.1 |
| 5 | 4.18 | 96 | 52 | 86 | 95.8 | 52 | 86.4 |
| 4 | 4.29 | 85 | 50 | 80 | 86 | 49.5 | 80 |
| 4 | 3.47 | 69 | 39 | 73 | 69.2 | 38.8 | 73.7 |
| 4 | 3.02 | 66 | 37 | 74 | 66.5 | 37.2 | 73.8 |
| 4 | 2.93 | 65 | 36 | 101 | 65 | 36 | 101 |
| 2 | 3.71 | 36 | 31 | 74 | 36 | 30.5 | 74 |
| 2 | 3.9 | 91 | 43 | 86 | 90.5 | 43.5 | 85.5 |
| 2 | 3.18 | 57 | 66 | 87 | 57 | 66.5 | 86.5 |
| 2 | 3.18 | 54 | 39 | 106 | 54.5 | 39 | 106 |
| 1 | 3.54 | 47 | 41 | 78 | 47 | 41 | 78 |
| 1 | 2.92 | 34 | 60 | 82 | 34 | 60 | 82 |
| 1 | 3.36 | 69 | 24 | 84 | 69 | 24 | 84 |
| 1 | 2.98 | 59 | 36 | 99 | 59 | 36 | 99 |

| **Males > Females during jaw movements in the male template space** | | | | | | | |
| --- | --- | --- | --- | --- | --- | --- | --- |
| Voxels | MAX | MAX X (vox) | MAX Y (vox) | MAX Z (vox) | COG X (vox) | COG Y (vox) | COG Z (vox) |
| 3040 | 4.8 | 59 | 60 | 85 | 65.8 | 44.3 | 89 |
| 1214 | 5.02 | 50 | 29 | 79 | 47.8 | 32.7 | 79.4 |
| 143 | 4.71 | 45 | 49 | 72 | 41.5 | 50.2 | 72.8 |
| 89 | 3.91 | 71 | 32 | 75 | 72 | 35 | 71.9 |
| 82 | 3.95 | 92 | 43 | 81 | 93 | 46.3 | 82.8 |
| 75 | 4.86 | 72 | 25 | 79 | 72.7 | 24.5 | 78 |
| 57 | 4.45 | 64 | 21 | 75 | 63.8 | 21.7 | 76.6 |
| 27 | 4.28 | 56 | 33 | 95 | 57.5 | 33.7 | 94 |
| 14 | 3.69 | 32 | 39 | 77 | 31.7 | 39.8 | 77.6 |
| 12 | 3.35 | 34 | 36 | 64 | 34.3 | 35.5 | 64.3 |
| 10 | 3.99 | 39 | 62 | 92 | 39.1 | 61.6 | 91.7 |
| 9 | 3.57 | 67 | 25 | 84 | 66.9 | 24.6 | 84.1 |
| 8 | 3.19 | 68 | 62 | 83 | 68.5 | 61.4 | 83.7 |
| 8 | 3.78 | 66 | 38 | 97 | 66.2 | 38.2 | 97.2 |
| 5 | 3.91 | 43 | 57 | 93 | 43.2 | 57.6 | 93.2 |
| 2 | 3.07 | 31 | 45 | 77 | 30.5 | 44.5 | 77 |
| 2 | 3.33 | 34 | 45 | 77 | 34 | 45.5 | 77 |
| 2 | 3.64 | 68 | 37 | 99 | 68 | 37.5 | 99 |
| 2 | 3.57 | 78 | 49 | 93 | 78 | 49.5 | 93 |
| 1 | 4.5 | 88 | 49 | 76 | 88 | 49 | 76 |
| 1 | 3.5 | 36 | 31 | 66 | 36 | 31 | 66 |
| 1 | 2.98 | 54 | 56 | 86 | 54 | 56 | 86 |
| 1 | 3.64 | 50 | 30 | 89 | 50 | 30 | 89 |
| 1 | 4.18 | 55 | 29 | 90 | 55 | 29 | 90 |

| **Males > Females during jaw movements in the study template space** | | | | | | | |
| --- | --- | --- | --- | --- | --- | --- | --- |
| Voxels | MAX | MAX X (vox) | MAX Y (vox) | MAX Z (vox) | COG X (vox) | COG Y (vox) | COG Z (vox) |
| 4772 | 4.92 | 42 | 39 | 97 | 59.2 | 42 | 91.6 |
| 156 | 3.95 | 40 | 61 | 99 | 43.3 | 61.4 | 96.9 |
| 45 | 4.73 | 44 | 39 | 77 | 42.4 | 36.6 | 76.7 |
| 33 | 3.94 | 41 | 53 | 93 | 40.5 | 52.8 | 93.6 |
| 29 | 4.24 | 85 | 50 | 80 | 88.7 | 48.8 | 79.9 |
| 24 | 3.66 | 89 | 47 | 86 | 90.3 | 44.7 | 85.7 |
| 20 | 4.48 | 55 | 31 | 98 | 52.9 | 31 | 97.2 |
| 17 | 4.16 | 70 | 33 | 81 | 69.4 | 32.8 | 79.6 |
| 16 | 3.85 | 96 | 52 | 86 | 95.9 | 51.5 | 86.6 |
| 13 | 3.13 | 67 | 32 | 101 | 68.5 | 33.6 | 99.3 |
| 8 | 3.42 | 93 | 49 | 89 | 92.5 | 49 | 89 |
| 8 | 3.4 | 36 | 63 | 93 | 36.8 | 62.8 | 92.6 |
| 8 | 3.41 | 41 | 69 | 93 | 41.5 | 68.6 | 92.6 |
| 6 | 3.12 | 57 | 66 | 87 | 58.3 | 65.8 | 87.2 |
| 6 | 3.92 | 73 | 38 | 75 | 72.5 | 37.2 | 75.5 |
| 4 | 3.55 | 66 | 26 | 90 | 66 | 26.5 | 90.5 |
| 3 | 2.72 | 34 | 43 | 93 | 34.3 | 43 | 93.7 |
| 2 | 3.27 | 66 | 52 | 82 | 66 | 51.5 | 82 |
| 2 | 3.87 | 51 | 59 | 92 | 51.4 | 59 | 92 |
| 1 | 3.23 | 62 | 54 | 82 | 62 | 54 | 82 |
| 1 | 3.12 | 95 | 49 | 82 | 95 | 49 | 82 |
| 1 | 3.04 | 93 | 49 | 77 | 93 | 49 | 77 |
| 1 | 3.38 | 69 | 39 | 73 | 69 | 39 | 73 |

Left Leg

None

Lips

| **Males > Females during lip movements in the female template space** | | | | | | | |
| --- | --- | --- | --- | --- | --- | --- | --- |
| Voxels | MAX | MAX X (vox) | MAX Y (vox) | MAX Z (vox) | COG X (vox) | COG Y (vox) | COG Z (vox) |
| 24 | 4.87 | 62 | 22 | 82 | 62.5 | 22.6 | 83.4 |

| **Males > Females during lip movements in the male template space** | | | | | | | |
| --- | --- | --- | --- | --- | --- | --- | --- |
| Voxels | MAX | MAX X (vox) | MAX Y (vox) | MAX Z (vox) | COG X (vox) | COG Y (vox) | COG Z (vox) |
| 26 | 4.88 | 64 | 22 | 78 | 63.5 | 21.6 | 76.5 |

| **Males > Females during lip movements in the study template space** | | | | | | | |
| --- | --- | --- | --- | --- | --- | --- | --- |
| Voxels | MAX | MAX X (vox) | MAX Y (vox) | MAX Z (vox) | COG X (vox) | COG Y (vox) | COG Z (vox) |
| 16 | 4.76 | 63 | 24 | 85 | 62.7 | 23.1 | 83.9 |

Right Leg

| **Females > males during lip movements in the female template space** | | | | | | | |
| --- | --- | --- | --- | --- | --- | --- | --- |
| Voxels | MAX | MAX X (vox) | MAX Y (vox) | MAX Z (vox) | COG X (vox) | COG Y (vox) | COG Z (vox) |
| 1377 | 4.42 | 93 | 44 | 64 | 90.1 | 58 | 64.2 |
| 442 | 4.34 | 34 | 59 | 61 | 37.8 | 56.8 | 64.3 |
| 95 | 3.94 | 88 | 40 | 75 | 86 | 36.9 | 73.1 |
| 27 | 3.86 | 87 | 31 | 76 | 85.7 | 30.2 | 76.7 |
| 21 | 4 | 92 | 34 | 80 | 91.9 | 34.7 | 78.4 |
| 9 | 4.37 | 82 | 27 | 59 | 82.1 | 26.8 | 59.1 |
| 8 | 4.28 | 36 | 42 | 62 | 35.6 | 42.4 | 62.4 |
| 8 | 3.66 | 42 | 65 | 64 | 42.4 | 65.5 | 64.1 |
| 6 | 4.22 | 41 | 43 | 72 | 41.3 | 42.5 | 72 |
| 5 | 3.72 | 88 | 39 | 70 | 88 | 39.2 | 70.4 |
| 4 | 4.24 | 81 | 25 | 61 | 81.5 | 24.5 | 61 |
| 3 | 4.17 | 36 | 36 | 62 | 36.3 | 35.7 | 62 |
| 2 | 3.88 | 31 | 62 | 61 | 31 | 62.5 | 61 |
| 2 | 3.61 | 32 | 66 | 65 | 32 | 65.5 | 65 |
| 1 | 3.36 | 46 | 59 | 58 | 46 | 59 | 58 |
| 1 | 4.05 | 83 | 72 | 63 | 83 | 72 | 63 |
| 1 | 3.69 | 84 | 72 | 65 | 84 | 72 | 65 |
| 1 | 3.3 | 92 | 67 | 66 | 92 | 67 | 66 |
| 1 | 3.22 | 91 | 70 | 57 | 91 | 70 | 57 |

| **Females > males during lip movements in the male template space** | | | | | | | |
| --- | --- | --- | --- | --- | --- | --- | --- |
| Voxels | MAX | MAX X (vox) | MAX Y (vox) | MAX Z (vox) | COG X (vox) | COG Y (vox) | COG Z (vox) |
| 1146 | 4.67 | 92 | 59 | 60 | 91.4 | 61.9 | 60.9 |
| 165 | 4.27 | 42 | 59 | 61 | 36.5 | 61.1 | 60.3 |
| 59 | 4.46 | 45 | 60 | 56 | 42.4 | 62.3 | 52.9 |
| 34 | 4.54 | 35 | 53 | 60 | 38.4 | 51.5 | 61.2 |
| 20 | 4.04 | 35 | 64 | 55 | 34.5 | 63 | 55.3 |
| 19 | 3.7 | 92 | 77 | 63 | 92 | 74.8 | 65.1 |
| 16 | 3.89 | 98 | 50 | 66 | 98.1 | 49 | 65.7 |
| 5 | 3.35 | 37 | 65 | 62 | 37.2 | 65.4 | 62 |
| 5 | 3.55 | 98 | 46 | 71 | 98.6 | 44.8 | 70.4 |
| 3 | 3.63 | 85 | 76 | 63 | 85.7 | 76.3 | 63 |
| 3 | 3.69 | 40 | 62 | 64 | 40 | 62.3 | 64.7 |
| 2 | 4.45 | 95 | 57 | 60 | 95.4 | 57 | 60 |
| 2 | 3.56 | 45 | 56 | 64 | 45 | 56 | 64.5 |
| 2 | 4.66 | 41 | 45 | 65 | 41 | 44.5 | 65 |
| 2 | 3.25 | 88 | 72 | 66 | 88 | 72.5 | 66 |
| 2 | 3.79 | 87 | 37 | 68 | 86.5 | 37 | 67.5 |
| 1 | 3.61 | 100 | 48 | 68 | 100 | 48 | 68 |

| **Females > males during lip movements in the study template space** | | | | | | | |
| --- | --- | --- | --- | --- | --- | --- | --- |
| Voxels | MAX | MAX X (vox) | MAX Y (vox) | MAX Z (vox) | COG X (vox) | COG Y (vox) | COG Z (vox) |
| 1044 | 4.25 | 93 | 44 | 64 | 89.7 | 58.3 | 63.5 |
| 28 | 4.02 | 42 | 53 | 67 | 41.6 | 54.8 | 66.9 |
| 21 | 3.93 | 32 | 56 | 65 | 32.5 | 56.1 | 65.4 |
| 17 | 4.31 | 95 | 46 | 69 | 96.1 | 47.2 | 69.4 |
| 17 | 3.68 | 40 | 58 | 56 | 40.5 | 58.8 | 57 |
| 16 | 4.34 | 45 | 56 | 61 | 44.4 | 56.1 | 60.7 |
| 14 | 3.82 | 37 | 62 | 64 | 36.1 | 60.7 | 64.3 |
| 12 | 3.68 | 91 | 71 | 66 | 90.1 | 70.3 | 66.9 |
| 8 | 3.99 | 85 | 36 | 72 | 85 | 36.2 | 72.7 |
| 6 | 4.23 | 34 | 59 | 61 | 34.2 | 58.8 | 60.5 |
| 6 | 3.74 | 38 | 48 | 68 | 38.5 | 48 | 68.3 |
| 5 | 3.76 | 39 | 60 | 54 | 38.4 | 59.6 | 54 |
| 4 | 3.6 | 92 | 67 | 56 | 92.2 | 66.8 | 55.5 |
| 3 | 3.68 | 31 | 59 | 64 | 31 | 59.3 | 64.3 |
| 3 | 3.68 | 33 | 65 | 60 | 33.7 | 64.3 | 61 |
| 2 | 3.27 | 97 | 45 | 63 | 96.5 | 45 | 63 |
| 2 | 4.05 | 92 | 34 | 80 | 92 | 34 | 79.5 |
| 1 | 3.76 | 45 | 54 | 63 | 45 | 54 | 63 |
| 1 | 3.94 | 32 | 68 | 60 | 32 | 68 | 60 |
| 1 | 4.07 | 84 | 33 | 75 | 84 | 33 | 75 |
| 1 | 3.67 | 89 | 35 | 75 | 89 | 35 | 75 |
| 1 | 4.01 | 87 | 31 | 76 | 87 | 31 | 76 |

Toes

| **Males > females during lip movements in the female template space** | | | | | | | |
| --- | --- | --- | --- | --- | --- | --- | --- |
| Voxels | MAX | MAX X (vox) | MAX Y (vox) | MAX Z (vox) | COG X (vox) | COG Y (vox) | COG Z (vox) |
| 8 | 6.45 | 62 | 21 | 82 | 61.8 | 21.5 | 82.4 |
| 1 | 4.98 | 61 | 24 | 86 | 61 | 24 | 86 |

| **Males > females during lip movements in the male template space** | | | | | | | |
| --- | --- | --- | --- | --- | --- | --- | --- |
| Voxels | MAX | MAX X (vox) | MAX Y (vox) | MAX Z (vox) | COG X (vox) | COG Y (vox) | COG Z (vox) |
| 10 | 6.29 | 63 | 21 | 75 | 62.7 | 20.5 | 75.1 |

| **Males > females during lip movements in the study template space** | | | | | | | |
| --- | --- | --- | --- | --- | --- | --- | --- |
| Voxels | MAX | MAX X (vox) | MAX Y (vox) | MAX Z (vox) | COG X (vox) | COG Y (vox) | COG Z (vox) |
| 10 | 6.45 | 62 | 22 | 82 | 62 | 21.8 | 82.5 |
| 3 | 4.51 | 61 | 21 | 78 | 60.7 | 21 | 78.7 |
| 2 | 4.9 | 61 | 24 | 87 | 61 | 24 | 86.5 |

Tongue

| **Males > females during tongue movements in the female template space** | | | | | | | |
| --- | --- | --- | --- | --- | --- | --- | --- |
| Voxels | MAX | MAX X (vox) | MAX Y (vox) | MAX Z (vox) | COG X (vox) | COG Y (vox) | COG Z (vox) |
| 1006 | 5.23 | 63 | 22 | 82 | 58.7 | 26.9 | 82.5 |
| 539 | 5.26 | 75 | 78 | 66 | 65.7 | 75.8 | 71 |
| 472 | 4.66 | 55 | 49 | 102 | 60.8 | 44.1 | 92.8 |
| 235 | 4.25 | 71 | 47 | 99 | 71.1 | 46.4 | 99.7 |
| 120 | 4.51 | 56 | 77 | 67 | 53 | 78.6 | 66.4 |
| 108 | 4.02 | 42 | 53 | 92 | 42.3 | 51.9 | 94.4 |
| 100 | 4.56 | 43 | 38 | 98 | 43.8 | 37 | 98.5 |
| 82 | 4.84 | 46 | 47 | 80 | 45.4 | 49.1 | 81.1 |
| 60 | 3.94 | 31 | 55 | 87 | 31.1 | 55 | 86.4 |
| 28 | 3.82 | 58 | 56 | 89 | 58.8 | 55.7 | 89 |
| 25 | 3.66 | 61 | 55 | 104 | 61.4 | 53 | 102 |
| 25 | 3.8 | 61 | 51 | 97 | 60.9 | 52.3 | 96.2 |
| 22 | 4.37 | 55 | 47 | 90 | 56 | 47 | 90.3 |
| 21 | 4.44 | 40 | 52 | 84 | 39.6 | 52.2 | 84.4 |
| 14 | 3.15 | 68 | 56 | 95 | 66.4 | 54.5 | 96.1 |
| 12 | 3.94 | 40 | 52 | 79 | 40.2 | 52.1 | 78.9 |
| 11 | 3.91 | 32 | 46 | 84 | 33 | 45.4 | 84 |
| 9 | 3.53 | 64 | 54 | 90 | 64.4 | 54 | 89.9 |
| 8 | 3.95 | 37 | 38 | 93 | 37 | 38.2 | 93.4 |
| 7 | 3.92 | 46 | 62 | 98 | 46 | 61.4 | 98.3 |
| 7 | 3.27 | 67 | 54 | 101 | 66.9 | 53.7 | 101 |
| 6 | 3.08 | 29 | 60 | 81 | 29.5 | 60.3 | 82.5 |
| 6 | 3.58 | 79 | 48 | 97 | 79 | 48 | 96.5 |
| 5 | 3.79 | 67 | 25 | 89 | 66.8 | 25.3 | 89.4 |
| 4 | 3.16 | 46 | 49 | 91 | 45.5 | 50 | 91 |
| 3 | 5.07 | 78 | 48 | 89 | 78 | 48 | 89 |
| 3 | 3.58 | 91 | 58 | 91 | 90.7 | 57.7 | 91 |
| 3 | 4.01 | 65 | 94 | 77 | 65 | 94.3 | 76.7 |
| 2 | 3.87 | 57 | 64 | 88 | 57.5 | 64 | 88 |
| 2 | 2.94 | 68 | 52 | 103 | 68 | 51.5 | 103 |
| 2 | 3.86 | 90 | 48 | 80 | 90.5 | 48 | 80 |
| 2 | 3.68 | 40 | 61 | 99 | 40 | 61 | 98.5 |
| 1 | 3.4 | 46 | 39 | 101 | 46 | 39 | 101 |
| 1 | 3.99 | 91 | 50 | 80 | 91 | 50 | 80 |
| 1 | 4.1 | 95 | 60 | 83 | 95 | 60 | 83 |
| 1 | 3.2 | 35 | 51 | 85 | 35 | 51 | 85 |
| 1 | 3.81 | 66 | 79 | 70 | 66 | 79 | 70 |
| 1 | 4.19 | 96 | 52 | 86 | 96 | 52 | 86 |
| 1 | 3.53 | 33 | 42 | 89 | 33 | 42 | 89 |
| 1 | 3.87 | 52 | 38 | 78 | 52 | 38 | 78 |

| **Males > females during tongue movements in the male template space** | | | | | | | |
| --- | --- | --- | --- | --- | --- | --- | --- |
| Voxels | MAX | MAX X (vox) | MAX Y (vox) | MAX Z (vox) | COG X (vox) | COG Y (vox) | COG Z (vox) |
| 1639 | 4.51 | 39 | 52 | 87 | 60.6 | 47.3 | 89.1 |
| 936 | 5.12 | 64 | 21 | 75 | 59.3 | 25.2 | 75.3 |
| 897 | 4.9 | 76 | 83 | 64 | 64 | 81 | 67.4 |
| 123 | 4.6 | 41 | 37 | 89 | 43.3 | 35.7 | 90.7 |
| 97 | 4.02 | 56 | 32 | 74 | 58 | 31.1 | 76.3 |
| 60 | 4.26 | 31 | 56 | 81 | 30.7 | 56.1 | 80.2 |
| 44 | 5.15 | 46 | 48 | 74 | 47.7 | 50.5 | 76 |
| 28 | 3.94 | 32 | 46 | 77 | 32.4 | 45.8 | 77 |
| 27 | 4.25 | 65 | 100 | 75 | 64.7 | 100 | 77.1 |
| 19 | 3.76 | 39 | 62 | 93 | 38.6 | 62.1 | 91.5 |
| 18 | 4.3 | 40 | 53 | 73 | 40.5 | 53.6 | 72.6 |
| 12 | 3.94 | 46 | 62 | 92 | 45.2 | 62.5 | 92.4 |
| 12 | 3.98 | 38 | 48 | 73 | 39.1 | 48.2 | 73.2 |
| 8 | 4.71 | 40 | 53 | 78 | 40 | 52.8 | 78.2 |
| 7 | 2.89 | 61 | 26 | 73 | 60.3 | 26.3 | 73.6 |
| 7 | 3.36 | 33 | 50 | 76 | 31.9 | 50.4 | 76.6 |
| 7 | 3.68 | 36 | 37 | 86 | 36.6 | 37.7 | 85.7 |
| 5 | 3.25 | 33 | 54 | 74 | 33.6 | 53.8 | 75 |
| 5 | 3.61 | 29 | 63 | 77 | 29 | 62.4 | 76.5 |
| 4 | 3.71 | 32 | 59 | 73 | 31.8 | 58.7 | 73.2 |
| 3 | 3.19 | 72 | 24 | 67 | 71.4 | 24.3 | 67 |
| 3 | 5.22 | 79 | 48 | 84 | 79 | 48 | 84 |
| 3 | 3.37 | 45 | 37 | 84 | 45 | 37 | 83 |
| 2 | 3.84 | 55 | 28 | 90 | 55 | 28.5 | 90 |
| 2 | 3.38 | 75 | 47 | 85 | 75 | 46.5 | 85 |
| 2 | 4.03 | 97 | 51 | 83 | 97 | 51.5 | 83 |
| 2 | 3.4 | 31 | 63 | 81 | 31.5 | 63 | 80.5 |
| 2 | 3.12 | 41 | 53 | 69 | 41 | 53 | 69.5 |
| 2 | 4.22 | 46 | 58 | 76 | 46 | 58 | 75.5 |
| 2 | 2.66 | 61 | 32 | 74 | 61 | 31.5 | 74 |
| 2 | 3.11 | 27 | 61 | 72 | 27.5 | 61 | 72 |
| 1 | 3.02 | 42 | 51 | 68 | 42 | 51 | 68 |
| 1 | 2.72 | 31 | 59 | 83 | 31 | 59 | 83 |
| 1 | 3.77 | 57 | 67 | 83 | 57 | 67 | 83 |
| 1 | 3.28 | 34 | 39 | 84 | 34 | 39 | 84 |
| 1 | 3.17 | 32 | 39 | 77 | 32 | 39 | 77 |
| 1 | 3.24 | 46 | 38 | 93 | 46 | 38 | 93 |
| 1 | 3.58 | 68 | 58 | 85 | 68 | 58 | 85 |
| 1 | 3.12 | 38 | 53 | 75 | 38 | 53 | 75 |
| 1 | 3.39 | 42 | 50 | 71 | 42 | 50 | 71 |

| **Males > females during tongue movements in the study template space** | | | | | | | |
| --- | --- | --- | --- | --- | --- | --- | --- |
| Voxels | MAX | MAX X (vox) | MAX Y (vox) | MAX Z (vox) | COG X (vox) | COG Y (vox) | COG Z (vox) |
| 1051 | 4.62 | 55 | 49 | 102 | 58.4 | 47.1 | 94.1 |
| 923 | 5.07 | 75 | 78 | 66 | 63.2 | 76.4 | 70 |
| 216 | 4.93 | 63 | 22 | 82 | 63.9 | 23.9 | 82.3 |
| 173 | 4.69 | 48 | 27 | 83 | 49.6 | 28 | 85.6 |
| 112 | 4.45 | 42 | 39 | 97 | 43.6 | 37.5 | 98.5 |
| 82 | 3.79 | 31 | 55 | 87 | 31.2 | 55.3 | 86.5 |
| 53 | 4.46 | 52 | 30 | 76 | 52 | 29.6 | 76.9 |
| 49 | 4.92 | 46 | 47 | 80 | 48.1 | 49.9 | 81.9 |
| 43 | 4.19 | 65 | 94 | 77 | 64.2 | 94.1 | 79.7 |
| 34 | 3.68 | 76 | 44 | 95 | 74.8 | 43.5 | 97.6 |
| 25 | 3.28 | 30 | 61 | 83 | 29.5 | 59.9 | 80.3 |
| 22 | 4.08 | 55 | 32 | 83 | 55.9 | 31.5 | 82.7 |
| 21 | 3.87 | 38 | 46 | 80 | 39.8 | 47.3 | 79.6 |
| 16 | 3.83 | 90 | 48 | 80 | 90.4 | 48.6 | 80.3 |
| 13 | 3.71 | 61 | 51 | 97 | 60.7 | 51.8 | 96.5 |
| 11 | 3.43 | 80 | 49 | 96 | 78.8 | 48.6 | 96.2 |
| 11 | 3.2 | 46 | 49 | 91 | 46 | 49.8 | 91.2 |
| 11 | 4.07 | 40 | 52 | 79 | 40.3 | 52.1 | 78.7 |
| 10 | 3.44 | 61 | 52 | 101 | 61.7 | 52.5 | 101 |
| 10 | 3.95 | 32 | 46 | 84 | 33.1 | 45.4 | 83.8 |
| 9 | 3.84 | 96 | 52 | 86 | 95.9 | 51.6 | 86.5 |
| 8 | 3.27 | 71 | 32 | 79 | 70.3 | 32 | 79.3 |
| 8 | 3.14 | 33 | 61 | 86 | 31.8 | 61 | 86.3 |
| 8 | 3.88 | 37 | 38 | 93 | 36.6 | 38.6 | 93.5 |
| 7 | 4.16 | 83 | 76 | 66 | 80.9 | 75.7 | 65.2 |
| 7 | 3.5 | 61 | 55 | 104 | 61.4 | 55.4 | 103 |
| 6 | 2.92 | 66 | 53 | 97 | 65.3 | 52.8 | 97.2 |
| 6 | 3.13 | 64 | 60 | 89 | 63.8 | 59.3 | 89 |
| 5 | 2.97 | 67 | 23 | 82 | 67 | 22.6 | 82.4 |
| 5 | 3.21 | 67 | 55 | 95 | 67.4 | 55.4 | 95.2 |
| 5 | 3.88 | 46 | 62 | 98 | 46.2 | 61.2 | 98.2 |
| 4 | 3.59 | 40 | 61 | 99 | 39.5 | 61 | 97.5 |
| 4 | 2.87 | 60 | 61 | 90 | 60.5 | 60.3 | 90.2 |
| 3 | 5.05 | 78 | 48 | 89 | 78.3 | 47.7 | 89 |
| 3 | 2.89 | 67 | 26 | 81 | 66.7 | 26.7 | 81 |
| 2 | 3.38 | 46 | 38 | 90 | 46 | 37.5 | 90 |
| 2 | 3.47 | 32 | 42 | 89 | 32.5 | 42 | 89 |
| 2 | 3.51 | 93 | 49 | 89 | 93 | 49 | 88.5 |
| 2 | 4.05 | 32 | 57 | 79 | 32 | 56.5 | 79 |
| 2 | 3.18 | 67 | 81 | 73 | 67 | 81 | 73.5 |
| 2 | 3.25 | 66 | 53 | 87 | 66.5 | 53 | 87 |
| 1 | 3.42 | 74 | 47 | 90 | 74 | 47 | 90 |
| 1 | 2.89 | 65 | 63 | 88 | 65 | 63 | 88 |
| 1 | 3.7 | 59 | 85 | 68 | 59 | 85 | 68 |
| 1 | 3.51 | 71 | 26 | 79 | 71 | 26 | 79 |
| 1 | 3.74 | 66 | 22 | 79 | 66 | 22 | 79 |
| 1 | 3.95 | 52 | 38 | 78 | 52 | 38 | 78 |
| 1 | 3.15 | 46 | 39 | 101 | 46 | 39 | 101 |

Upper arms

| **Males > females during upper arm movements in the female template space** | | | | | | | |
| --- | --- | --- | --- | --- | --- | --- | --- |
| Voxels | MAX | MAX X (vox) | MAX Y (vox) | MAX Z (vox) | COG X (vox) | COG Y (vox) | COG Z (vox) |
| 171 | 5.14 | 34 | 64 | 79 | 34.9 | 61.9 | 84.3 |
| 43 | 4.88 | 79 | 75 | 77 | 79.5 | 74.1 | 77.7 |
| 32 | 4.71 | 72 | 69 | 81 | 72 | 68.4 | 80.5 |
| 18 | 4.11 | 91 | 62 | 81 | 91.2 | 60.5 | 81.9 |
| 14 | 4.56 | 95 | 63 | 77 | 94.4 | 63.4 | 77.4 |
| 10 | 5.33 | 91 | 72 | 78 | 90.4 | 71.9 | 77.9 |
| 4 | 3.85 | 80 | 65 | 77 | 80.3 | 65.5 | 77.2 |
| 4 | 3.82 | 67 | 65 | 81 | 67 | 65.5 | 81 |
| 2 | 4.57 | 72 | 75 | 77 | 72 | 75 | 77.5 |
| 1 | 4.21 | 48 | 58 | 72 | 48 | 58 | 72 |
| 1 | 3.99 | 75 | 65 | 76 | 75 | 65 | 76 |
| 1 | 4.23 | 46 | 60 | 79 | 46 | 60 | 79 |
| 1 | 4.08 | 46 | 64 | 79 | 46 | 64 | 79 |
| 1 | 4.69 | 70 | 62 | 81 | 70 | 62 | 81 |
| 1 | 4.88 | 76 | 71 | 81 | 76 | 71 | 81 |

| **Males > females during upper arm movements in the male template space** | | | | | | | |
| --- | --- | --- | --- | --- | --- | --- | --- |
| Voxels | MAX | MAX X (vox) | MAX Y (vox) | MAX Z (vox) | COG X (vox) | COG Y (vox) | COG Z (vox) |
| 302 | 5.68 | 34 | 67 | 74 | 34.5 | 63.9 | 79.2 |
| 211 | 4.89 | 80 | 79 | 75 | 77.6 | 72.5 | 75.9 |
| 29 | 4.64 | 96 | 66 | 74 | 95 | 65.1 | 76.1 |
| 11 | 4.13 | 93 | 60 | 80 | 91.8 | 60.5 | 79.7 |
| 4 | 4.36 | 46 | 62 | 74 | 46.5 | 62.5 | 74 |
| 3 | 4.08 | 49 | 60 | 68 | 49 | 60.7 | 67.7 |
| 1 | 4.9 | 73 | 79 | 75 | 73 | 79 | 75 |
| 1 | 5.57 | 92 | 75 | 76 | 92 | 75 | 76 |

| **Males > females during upper arm movements in the study template space** | | | | | | | |
| --- | --- | --- | --- | --- | --- | --- | --- |
| Voxels | MAX | MAX X (vox) | MAX Y (vox) | MAX Z (vox) | COG X (vox) | COG Y (vox) | COG Z (vox) |
| 201 | 5.26 | 34 | 64 | 79 | 34.7 | 61.9 | 84.6 |
| 51 | 4.59 | 95 | 63 | 77 | 92.6 | 61.9 | 79.9 |
| 48 | 4.91 | 79 | 75 | 77 | 79.5 | 73.9 | 77.7 |
| 40 | 4.53 | 72 | 69 | 81 | 72 | 68.1 | 80.5 |
| 18 | 3.99 | 81 | 64 | 77 | 80.7 | 66.1 | 77.3 |
| 9 | 5.55 | 91 | 72 | 78 | 90.3 | 71.9 | 78 |
| 2 | 4.27 | 72 | 75 | 77 | 72 | 75 | 77.5 |
| 1 | 3.93 | 75 | 65 | 76 | 75 | 65 | 76 |
| 1 | 4.52 | 46 | 60 | 79 | 46 | 60 | 79 |
| 1 | 4.95 | 70 | 62 | 81 | 70 | 62 | 81 |
| 1 | 4.67 | 76 | 71 | 81 | 76 | 71 | 81 |

Wrists

| **Males > females during wrist movements in the female template space** | | | | | | | |
| --- | --- | --- | --- | --- | --- | --- | --- |
| Voxels | MAX | MAX X (vox) | MAX Y (vox) | MAX Z (vox) | COG X (vox) | COG Y (vox) | COG Z (vox) |
| 9764 | 5.57 | 76 | 70 | 102 | 65.5 | 71.7 | 84.6 |
| 3123 | 5.19 | 62 | 21 | 82 | 70.8 | 34.7 | 86.3 |
| 358 | 5.08 | 38 | 37 | 81 | 41.1 | 34 | 83.4 |
| 137 | 3.71 | 67 | 48 | 101 | 71 | 47.9 | 98.6 |
| 102 | 4.72 | 42 | 38 | 97 | 44.2 | 36.7 | 98.2 |
| 65 | 3.73 | 51 | 81 | 71 | 52.7 | 80.9 | 69.1 |
| 57 | 3.58 | 74 | 41 | 76 | 73.5 | 42.6 | 77.2 |
| 22 | 4.53 | 69 | 47 | 70 | 69.1 | 46.8 | 69.8 |
| 16 | 3.7 | 52 | 27 | 87 | 52.1 | 26.3 | 87.5 |
| 14 | 3.71 | 49 | 51 | 68 | 48.3 | 52 | 67.2 |
| 10 | 3.17 | 97 | 45 | 88 | 96.5 | 44.6 | 86.8 |
| 9 | 3.53 | 51 | 30 | 86 | 50.6 | 30.6 | 86 |
| 8 | 3.08 | 72 | 64 | 91 | 71.6 | 64.5 | 91.8 |
| 5 | 4.02 | 54 | 62 | 83 | 54 | 61.4 | 82.6 |
| 5 | 3.3 | 63 | 64 | 83 | 63.6 | 63.4 | 82.8 |
| 5 | 4.28 | 38 | 39 | 54 | 37.8 | 39.6 | 53.8 |
| 4 | 3.15 | 79 | 41 | 81 | 79.2 | 41 | 80.2 |
| 4 | 3.48 | 58 | 56 | 90 | 59 | 56 | 89.8 |
| 3 | 3.17 | 62 | 64 | 91 | 61.4 | 64 | 91.3 |
| 3 | 3.13 | 55 | 31 | 98 | 54.3 | 31 | 98.3 |
| 3 | 3.93 | 72 | 40 | 82 | 72 | 40.3 | 82.3 |
| 3 | 3.82 | 52 | 66 | 83 | 51.7 | 66 | 82.7 |
| 3 | 4.04 | 77 | 78 | 68 | 77 | 77.7 | 67.3 |
| 3 | 4.05 | 87 | 52 | 73 | 86.7 | 52 | 73.3 |
| 2 | 2.88 | 67 | 45 | 96 | 67.5 | 45 | 96 |
| 2 | 2.81 | 48 | 82 | 72 | 47.5 | 82 | 72 |
| 2 | 3.75 | 75 | 46 | 91 | 75.5 | 46 | 91 |
| 2 | 3.11 | 65 | 62 | 90 | 65 | 61.5 | 90 |
| 2 | 2.86 | 63 | 65 | 89 | 62.5 | 64.5 | 89 |
| 2 | 2.17 | 49 | 48 | 77 | 48.5 | 48 | 77 |
| 2 | 2.66 | 78 | 63 | 85 | 77.5 | 63 | 85 |
| 2 | 2.64 | 84 | 86 | 84 | 84 | 85.5 | 84 |
| 2 | 3.1 | 43 | 72 | 97 | 42.5 | 72 | 97 |
| 1 | 3.2 | 51 | 65 | 88 | 51 | 65 | 88 |
| 1 | 2.47 | 82 | 41 | 81 | 82 | 41 | 81 |
| 1 | 2.62 | 74 | 59 | 85 | 74 | 59 | 85 |
| 1 | 2.89 | 34 | 52 | 81 | 34 | 52 | 81 |
| 1 | 3.24 | 71 | 62 | 92 | 71 | 62 | 92 |
| 1 | 2.83 | 67 | 47 | 94 | 67 | 47 | 94 |
| 1 | 3.38 | 57 | 65 | 64 | 57 | 65 | 64 |
| 1 | 3.75 | 45 | 58 | 64 | 45 | 58 | 64 |
| 1 | 3.93 | 80 | 63 | 84 | 80 | 63 | 84 |

| **Males > females during wrist movements in the male template space** | | | | | | | |
| --- | --- | --- | --- | --- | --- | --- | --- |
| Voxels | MAX | MAX X (vox) | MAX Y (vox) | MAX Z (vox) | COG X (vox) | COG Y (vox) | COG Z (vox) |
| 6595 | 5.54 | 69 | 80 | 81 | 70.7 | 80 | 85.4 |
| 3918 | 5.37 | 66 | 24 | 82 | 70.7 | 33.9 | 79.6 |
| 1493 | 4.99 | 46 | 56 | 69 | 44.1 | 64.9 | 72.5 |
| 942 | 4.81 | 38 | 38 | 74 | 42.1 | 38.6 | 75.5 |
| 733 | 4.95 | 79 | 65 | 68 | 76.7 | 62.2 | 71.4 |
| 242 | 3.75 | 67 | 47 | 95 | 71.3 | 47 | 92.3 |
| 94 | 4 | 54 | 87 | 64 | 52.9 | 86.3 | 66.3 |
| 85 | 3.82 | 44 | 78 | 74 | 42 | 78.3 | 74.5 |
| 47 | 3.41 | 80 | 66 | 74 | 81.5 | 68 | 74.2 |
| 46 | 3.17 | 62 | 66 | 86 | 63.4 | 65.3 | 85.6 |
| 39 | 4.12 | 55 | 77 | 77 | 58.4 | 74.6 | 76 |
| 29 | 4.8 | 41 | 37 | 89 | 42.9 | 35.3 | 89.9 |
| 25 | 4.26 | 97 | 51 | 84 | 96.1 | 50.5 | 83.9 |
| 21 | 4.75 | 78 | 83 | 65 | 77.7 | 82.4 | 64.9 |
| 21 | 3.25 | 98 | 44 | 83 | 97 | 42.9 | 83 |
| 11 | 3.14 | 51 | 67 | 84 | 50.9 | 67.4 | 83.6 |
| 11 | 3.04 | 75 | 92 | 81 | 75.2 | 92.9 | 80.6 |
| 11 | 4.42 | 38 | 45 | 47 | 38.4 | 43.8 | 47.5 |
| 11 | 3.59 | 32 | 70 | 84 | 32.5 | 70.4 | 84.5 |
| 10 | 3.21 | 75 | 79 | 73 | 74.2 | 78.7 | 74.4 |
| 9 | 3.33 | 63 | 69 | 74 | 63.3 | 67.8 | 74.4 |
| 8 | 3.44 | 84 | 70 | 64 | 83.5 | 70 | 64.2 |
| 8 | 2.92 | 82 | 66 | 69 | 82.7 | 66.5 | 69 |
| 8 | 3.32 | 97 | 35 | 80 | 96.4 | 35.5 | 80.6 |
| 7 | 2.7 | 74 | 84 | 88 | 74 | 82.8 | 87.7 |
| 7 | 2.8 | 61 | 45 | 66 | 61.1 | 44.3 | 66.7 |
| 7 | 3.01 | 34 | 54 | 75 | 33.7 | 54.1 | 74.7 |
| 6 | 3.85 | 56 | 80 | 74 | 55.7 | 80 | 74.5 |
| 6 | 4.4 | 58 | 71 | 78 | 58.2 | 70.5 | 77.8 |
| 5 | 3.94 | 56 | 27 | 48 | 56.4 | 27.8 | 48 |
| 5 | 2.83 | 64 | 75 | 67 | 63.8 | 75.4 | 67.6 |
| 4 | 4.04 | 73 | 74 | 77 | 72.8 | 73.8 | 76.3 |
| 4 | 4.6 | 43 | 37 | 45 | 43.5 | 36.5 | 45 |
| 4 | 4.09 | 40 | 48 | 48 | 39.3 | 48.5 | 47.8 |
| 3 | 5.07 | 54 | 30 | 47 | 54 | 30 | 47 |
| 3 | 4.14 | 89 | 54 | 69 | 88.7 | 54 | 69.3 |
| 2 | 3.52 | 76 | 46 | 86 | 76 | 45.5 | 86 |
| 2 | 2.83 | 70 | 46 | 85 | 69.5 | 46 | 85 |
| 2 | 4.92 | 63 | 34 | 52 | 63 | 33.5 | 52 |
| 2 | 3.79 | 62 | 79 | 68 | 62 | 79.5 | 68 |
| 2 | 3.47 | 66 | 73 | 80 | 66.5 | 73 | 80 |
| 2 | 2.48 | 43 | 25 | 78 | 42.5 | 25 | 78 |
| 2 | 2.54 | 57 | 98 | 77 | 56.5 | 98 | 77 |
| 2 | 2.91 | 66 | 74 | 77 | 66 | 73.5 | 77 |
| 2 | 3.13 | 61 | 43 | 72 | 61.5 | 43.5 | 72.5 |
| 2 | 2.53 | 97 | 67 | 71 | 97.5 | 67.5 | 71 |
| 2 | 3.11 | 77 | 67 | 93 | 77 | 67 | 92.5 |
| 1 | 2.9 | 44 | 40 | 74 | 44 | 40 | 74 |
| 1 | 3.01 | 56 | 81 | 91 | 56 | 81 | 91 |
| 1 | 3.03 | 73 | 44 | 99 | 73 | 44 | 99 |
| 1 | 4.06 | 69 | 30 | 46 | 69 | 30 | 46 |
| 1 | 2.73 | 48 | 41 | 73 | 48 | 41 | 73 |
| 1 | 2.39 | 80 | 56 | 87 | 80 | 56 | 87 |
| 1 | 3.36 | 65 | 68 | 71 | 65 | 68 | 71 |
| 1 | 2.86 | 69 | 43 | 84 | 69 | 43 | 84 |
| 1 | 2.47 | 61 | 102 | 70 | 61 | 102 | 70 |
| 1 | 3.34 | 79 | 76 | 82 | 79 | 76 | 82 |
| 1 | 2.35 | 70 | 87 | 66 | 70 | 87 | 66 |
| 1 | 3.37 | 82 | 67 | 77 | 82 | 67 | 77 |
| 1 | 3.34 | 77 | 67 | 70 | 77 | 67 | 70 |
| 1 | 2.95 | 35 | 56 | 70 | 35 | 56 | 70 |
| 1 | 3.44 | 75 | 74 | 78 | 75 | 74 | 78 |
| 1 | 2.88 | 69 | 74 | 77 | 69 | 74 | 77 |
| 1 | 3.9 | 41 | 40 | 49 | 41 | 40 | 49 |

| **Males > females during wrist movements in the study template space** | | | | | | | |
| --- | --- | --- | --- | --- | --- | --- | --- |
| Voxels | MAX | MAX X (vox) | MAX Y (vox) | MAX Z (vox) | COG X (vox) | COG Y (vox) | COG Z (vox) |
| 7754 | 5.52 | 76 | 70 | 102 | 71 | 74.9 | 86.2 |
| 2674 | 4.91 | 76 | 25 | 86 | 73.1 | 32.4 | 85.2 |
| 2486 | 5.11 | 46 | 54 | 74 | 43.4 | 52.8 | 79.3 |
| 331 | 4.61 | 61 | 51 | 88 | 61.5 | 44 | 87.4 |
| 152 | 4.82 | 59 | 47 | 98 | 56.2 | 47.6 | 98.7 |
| 131 | 4.7 | 42 | 38 | 97 | 44.4 | 37 | 98.6 |
| 121 | 3.56 | 73 | 46 | 99 | 71 | 48 | 98.5 |
| 76 | 3.67 | 51 | 83 | 70 | 52.5 | 81 | 69.2 |
| 75 | 3.24 | 41 | 60 | 99 | 42.5 | 60 | 98.8 |
| 34 | 3.19 | 97 | 45 | 88 | 95.8 | 43.5 | 87 |
| 33 | 3.73 | 49 | 51 | 68 | 48.2 | 52 | 67.4 |
| 16 | 3.63 | 82 | 66 | 67 | 82.3 | 65.9 | 66.7 |
| 16 | 3.63 | 44 | 73 | 79 | 44.4 | 73.6 | 79 |
| 10 | 3.14 | 61 | 64 | 91 | 62.2 | 64.5 | 89.7 |
| 10 | 3.04 | 80 | 56 | 96 | 80.2 | 56.5 | 95.5 |
| 9 | 4.5 | 77 | 78 | 67 | 76.7 | 77.1 | 66.8 |
| 8 | 3.08 | 55 | 31 | 98 | 54.4 | 30.9 | 97.8 |
| 8 | 4.3 | 37 | 41 | 53 | 37.6 | 40.2 | 53.7 |
| 7 | 3.04 | 63 | 64 | 83 | 64.1 | 63.8 | 82.6 |
| 7 | 3.21 | 95 | 36 | 85 | 94.5 | 37 | 85.5 |
| 4 | 3.33 | 58 | 56 | 90 | 59 | 56 | 89.7 |
| 4 | 4.5 | 69 | 47 | 70 | 69 | 46.8 | 70 |
| 4 | 3.46 | 40 | 70 | 85 | 40.7 | 70.2 | 85 |
| 3 | 3.85 | 74 | 58 | 88 | 74.3 | 57.7 | 88 |
| 3 | 3.41 | 39 | 72 | 80 | 39 | 72.9 | 80 |
| 2 | 3.08 | 65 | 62 | 90 | 65 | 61.5 | 90 |
| 2 | 3 | 47 | 48 | 65 | 46.5 | 48 | 65 |
| 2 | 3.39 | 75 | 46 | 91 | 75.5 | 46 | 91 |
| 2 | 3.02 | 85 | 47 | 76 | 85 | 46.5 | 76 |
| 2 | 3.19 | 73 | 44 | 76 | 73 | 43.5 | 76 |
| 2 | 3.68 | 74 | 41 | 76 | 74.5 | 41 | 76 |
| 2 | 2.87 | 52 | 54 | 66 | 52 | 53.5 | 66 |
| 1 | 4.06 | 45 | 46 | 91 | 45 | 46 | 91 |
| 1 | 3.46 | 71 | 61 | 92 | 71 | 61 | 92 |
| 1 | 2.65 | 45 | 64 | 101 | 45 | 64 | 101 |
| 1 | 2.85 | 89 | 49 | 86 | 89 | 49 | 86 |
| 1 | 2.6 | 75 | 87 | 83 | 75 | 87 | 83 |
| 1 | 2.67 | 42 | 70 | 82 | 42 | 70 | 82 |
| 1 | 3.14 | 85 | 80 | 80 | 85 | 80 | 80 |
| 1 | 3.58 | 69 | 45 | 76 | 69 | 45 | 76 |
| 1 | 2.44 | 87 | 69 | 75 | 87 | 69 | 75 |
| 1 | 4.34 | 68 | 83 | 73 | 68 | 83 | 73 |
| 1 | 4.28 | 87 | 52 | 73 | 87 | 52 | 73 |
| 1 | 4.1 | 54 | 51 | 67 | 54 | 51 | 67 |
| 1 | 4.69 | 61 | 32 | 58 | 61 | 32 | 58 |
| 1 | 3.44 | 43 | 72 | 97 | 43 | 72 | 97 |
